# Host Oxidative Response Capacity Determines Longevity Outcomes of Microbial Interventions

**DOI:** 10.1111/acel.70418

**Published:** 2026-02-12

**Authors:** Xusheng Hao, Rongwei Yuan, Yafei Guo, Guanyu Chen, Yongqing Guo, Limeng Liu, Fei Lu, Yang Bai, Ye Tian

**Affiliations:** ^1^ Institute of Genetics and Developmental Biology Chinese Academy of Sciences Beijing China; ^2^ University of Chinese Academy of Sciences Beijing China; ^3^ Laboratory of Advanced Breeding Technologies, Institute of Genetics and Developmental Biology Chinese Academy of Sciences Beijing China; ^4^ CAS‐JIC Centre of Excellence for Plant and Microbial Science (CEPAMS), Institute of Genetics and Developmental Biology Chinese Academy of Sciences Beijing China; ^5^ State Key Laboratory for Gene Function and Modulation Research, New Cornerstone Science Laboratory, Peking‐Tsinghua Center for Life Sciences, School of Life Sciences Peking University Beijing China

**Keywords:** CRISPR‐Cas9 allelic recapitulation, genetic variation, host‐microbial interventions, longevity, oxidative stress resilience

## Abstract

Microbial communities profoundly influence host aging, yet how natural genetic variation determines microbiota‐driven longevity remains unclear. By screening root‐derived bacterial isolates across genetically diverse *Caenorhabditis elegans* strains, we identified striking phenotypic heterogeneity, ranging from lifespan extension to accelerated aging. Combining classical genetic analysis, quantitative trait locus (QTL) mapping and CRISPR‐Cas9 allelic recapitulation, we identify *skn‐1* (Nrf2) and *gsy‐1* (glycogen synthase) as key host determinants. We demonstrate that strains with mutations or specific natural variants in these loci exhibit a compromised redox buffering capacity, leading to systemic oxidative stress, loss of tissue integrity, and premature death upon microbial challenge. Conversely, robust hosts utilize the same microbial signals to promote longevity. Notably, lifespan defects in susceptible individuals were rescued by antioxidant supplementation. These findings establish redox homeostasis as a central axis in host‐microbe‐aging interactions and provide a mechanistic framework for precision microbiome interventions tailored to host genetic backgrounds.

## Graphical abstract

Genetic variation determines whether microbial interactions extend or shorten host lifespan via redox homeostasis. This discovery highlights the critical role of host genetics in shaping microbiota‐driven aging, offering a new framework for precision microbiome interventions tailored to individual needs for promoting longevity.

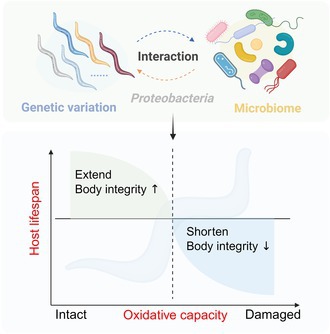

## 1 Introduction

Aging is a multifactorial process shaped by the interplay of genetic, nutrient, environmental, and microbial factors, making it essential to understand the interactions among these components to develop effective interventions (Lopez‐Otin et al. 2013, 2023). Longevity‐promoting strategies, such as dietary restriction, genetic manipulation, and pharmacological treatments, have been extensively studied in model organisms including *Caenorhabditis elegans*, *Drosophila*, and mice (Apfeld and Kenyon 1998; Cabreiro et al. 2013; Robida‐Stubbs et al. 2012; Piper et al. 2011; Greer et al. 2007; Backes et al. 2021; Friedman and Johnson 1988; Eisenberg et al. 2009; Gao et al. 2024). Among these factors, the microbiome has emerged as a critical and dynamic regulator of host aging. Comprising tens of thousands of microbial species (Nicholson et al. 2005; Dodd et al. 2017), the microbiome influences host physiology through its effects on metabolism, immunity, and neural function (Gomaa 2020; Ley et al. 2006; Ducarmon et al. 2019; Fan and Pedersen 2021). Disruptions in microbiota composition, or dysbiosis, have been linked to a range of age‐related conditions, including chronic inflammation, metabolic dysfunction, and neurodegeneration (Ghosh et al. 2022; Claesson et al. 2012; Biagi et al. 2016). These associations have promoted growing interest in microbiome‐targeted interventions such as probiotics, prebiotics, and rationally selected bacteria consortia, to delay age‐related decline and extend healthspan (Ghosh et al. 2022; Barcena et al. 2019; Fukuda et al. 2012; Suez et al. 2019).

However, most studies of microbiota‐based aging interventions have relied on genetically homogeneous host models, which fail to account for the genetic variation present in natural populations. This is a significant limitation, as host genetic background can significantly influence responses to microbial interventions (Pang and Curran 2014; Zhang, Weckhorst, et al. 2021). Certain genetic variants may enhance or impair the beneficial effects of microbiome interventions, or even lead to deleterious outcomes. The complexity of microbial communities and the vast diversity of host genotypes pose major challenges for studying these interactions in mammalian systems, where large‐scale, controlled testing is logistically and ethically constrained. Thus, how natural genetic variation modulates microbiota‐induced longevity remains largely unresolved.

*Caenorhabditis elegans* provides a powerful platform for investigating host–microbe interactions in aging due to its short lifespan, tractable genetics, and modifiable microbial environment (Watson et al. 2014; Zhang et al. 2019; Qi and Han 2018; O'Donnell et al. 2020; Samuel et al. 2016). Although studies in this model have uncovered key aging pathways (Han et al. 2017; Donato et al. 2017; Bhat et al. 2024; Shin et al. 2020; Sonowal et al. 2017), they have largely been confined to isogenic strains, limiting our understanding of how genetic diversity affects microbial influences on aging. Here, we leveraged a genetically diverse *C. elegans* population to assess longevity responses to microbial interventions. Specifically, we focused on bacterial isolates from *Arabidopsis* roots (Bai et al. 2015; Wang et al. 2024), an ecologically relevant microbiota source with defined composition and prior utility in dissecting host–microbe interactions.

We systemically evaluated the lifespan of eight representative bacterial genera from the phyla *Proteobacteria* and *Actinobacteria* across five genetically distinct *C. elegans* strains. Our results uncovered a profound genetic‐context dependency: specific microbes that promoted longevity in certain backgrounds acted as potent accelerators of aging in others. A prominent example is *Variovorax* sp. Root473, which extended the lifespan of wild‐type animals but significantly shortened the survival of *skn‐1* mutants, which lack robust oxidative stress defenses. This deleterious effect was mechanistically linked to a breakdown in redox homeostasis and a systemic loss of body integrity, both of which were fully reversible through exogenous antioxidant supplementation.

To decipher the broader genetic architecture underlying these interactions, we expanded our screen to include 38 natural isolates and a mapping population of 36 recombinant inbred lines (RILs). This quantitative approach, followed by precise CRISPR‐Cas9 validation, identified *gsy‐1* (encoding glycogen synthase) as a critical modifier of microbe‐induced aging. We demonstrate that, similar to loss of *skn‐1*, *gsy‐1* deficiency compromises the host's oxidative buffering capacity, rendering the animal hypersensitive to specific microbial exposure.

Together, our finding reveals that host genetic variation dictates the “tipping point” between microbial benefit and toxicity by modulating oxidative stress resilience. This work underscores the need to integrate host genetic diversity into microbiome‐aging research and establishes a mechanistic foundation for developing precision microbiome therapies tailored to individual genotypes.

## 2 Results

### 2.1 Host Genetic–Microbiome Interaction Screening Reveals Lifespan Heterogeneity

To systematically investigate how host genetic variation shapes microbial influence on longevity, we performed a large‐scale screen in *C. elegans* using eight representative bacterial isolates from the *Arabidopsis* root microbiota collection (Table S1) (Bai et al. 2015). These isolates, primarily from the *Proteobacteria* and *Actinobacteria* phyla, were selected based on their previously reported capacity to extend the lifespan of wild‐type N2 animals (Liu et al. 2024).

**FIGURE 1:**
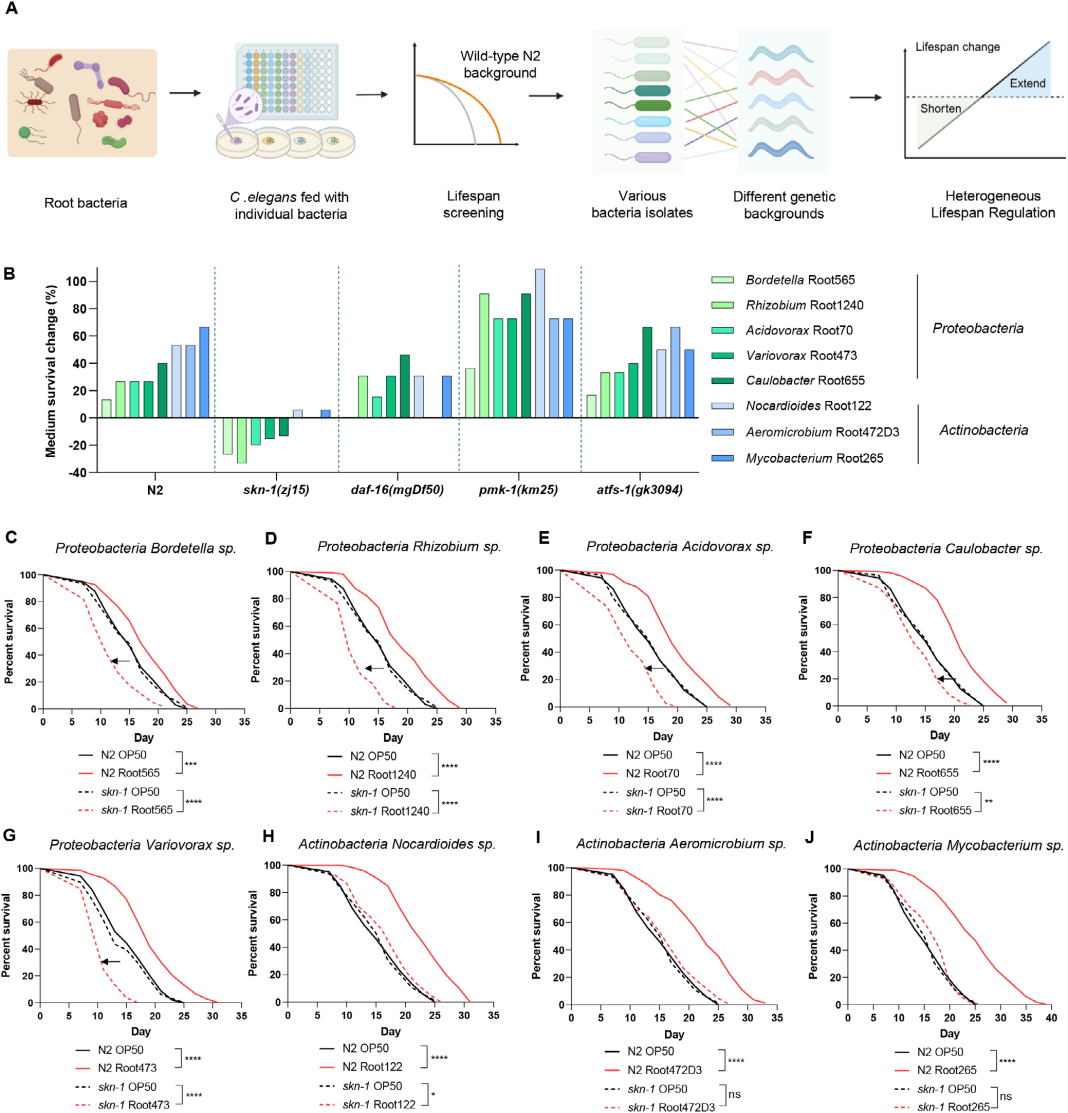
Host genetic background dictates the longevity outcomes of root‐derived microbial isolates. (A) Experimental design for lifespan screening: The study screened 119 *Arabidopsis* root‐derived bacterial species from the root microbiome for their lifespan effects on *Caenorhabditis elegans*. Individual bacteria were cultured and seeded on NGM plates and synchronized wild‐type nematodes (N2) were then exposed to each bacterium for lifespan assessment. Our previous study has found that bacteria from eight taxonomic genera can extend the lifespan of wild‐type animals N2. Here, we investigated the lifespan effect of these eight bacterial isolates on multiple *C. elegans* genetic backgrounds, including wild‐type N2, *skn‐1(zj15)*, *daf‐16(mgDf50)*, *pmk‐1(km25)*, and *atfs‐1(gk3094)*, with *Escherichia coli* OP50 as a control (diagram created in BioRender). (B) Lifespan effects of different host genetic backgrounds with specific bacterial isolates. Lifespan changes relative to the *E. coli* OP50 control are shown for *Proteobacteria* species (green) and *Actinobacteria* species (blue). The vertical axis indicates the percentage change in medium survival. (C) Lifespan analysis of WT and *skn‐1(zj15)* animals fed with OP50 and *Bordetella* sp. Root565. (D) Lifespan analysis of WT and *skn‐1(zj15)* animals fed with OP50 and *Rhizobium* sp. Root1240. (E) Lifespan analysis of WT and *skn‐1(zj15)* animals fed with OP50 and *Acidovorax* sp. Root70. (F) Lifespan analysis of WT and *skn‐1(zj15)* animals fed with OP50 and *Caulobacter* sp. Root655. (G) Lifespan analysis of N2 and *skn‐1(zj15)* animals fed with OP50 and *Variovorax* sp. Root473. (H) Lifespan analysis of WT and *skn‐1(zj15)* animals fed with OP50 and *Nocardioides* sp. Root122. (I) Lifespan analysis of WT and *skn‐1(zj15)* animals fed with OP50 and *Aeromicrobium* sp. Root472D3. (J) Lifespan analysis of WT and *skn‐1(zj15)* animals fed with OP50 and *Mycobacterium* sp. Root265. ****p < 0.0001, ***p < 0.001, **p < 0.01, *p < 0.05, ns denotes *p* > 0.05 via log‐rank (Mantel–Cox) test. Source data are provided in Table S10.

We evaluated these bacterial strains across five distinct backgrounds: wild‐type (N2) animals and four mutant strains lacking conserved aging regulators: *skn‐1* (oxidative stress transcription factor) (Robida‐Stubbs et al. 2012; Blackwell et al. 2015; Tullet et al. 2008), *daf‐16* (the FOXO transcription factor) (Henderson and Johnson 2001; Lee et al. 2001; Lin et al. 2001), *pmk‐1* (the p38 MAP kinase) (Troemel et al. 2006), and *atfs‐1* (the mitochondrial unfolded protein response regulator) (Zhang et al. 2018; Nargund et al. 2012) (Figure 1A). Across 45 independent lifespan assays (*n* ≥ 100 individuals per group; Table S2), we observed that microbial effects on longevity were highly genotype‐dependent (Figure 1B). For instance, while certain isolates extended lifespan more robustly in *pmk‐1* mutants, *atfs‐1* mutants largely mirrored the wild‐type response (Figure 1B). *daf‐16* was partially required for the pro‐longevity effects of specific bacterial strains, suggesting an interplay between the microbiota and the insulin/IGF‐1 signaling pathway (Figure 1B).

Notably, several *Proteobacteria* isolates that extended lifespan in wild‐type animals significantly accelerated aging in *skn‐1* mutants (Figure 1B–G). In contrast, *Actinobacteria* isolates elicited neutral or minimal effects in the *skn‐1* mutant background (Figure 1H–J). This divergent response identifies *skn‐1* as a critical gatekeeper of microbial influence on aging. To dissect this further, we focused on *Variovorax* sp. Root473 in subsequent analyses, a *Proteobacteria* strain with divergent lifespan effects.

### 2.2 Natural Genetic Variations Modulate Lifespan Response to *Variovorax* sp. Root473

**FIGURE 2:**
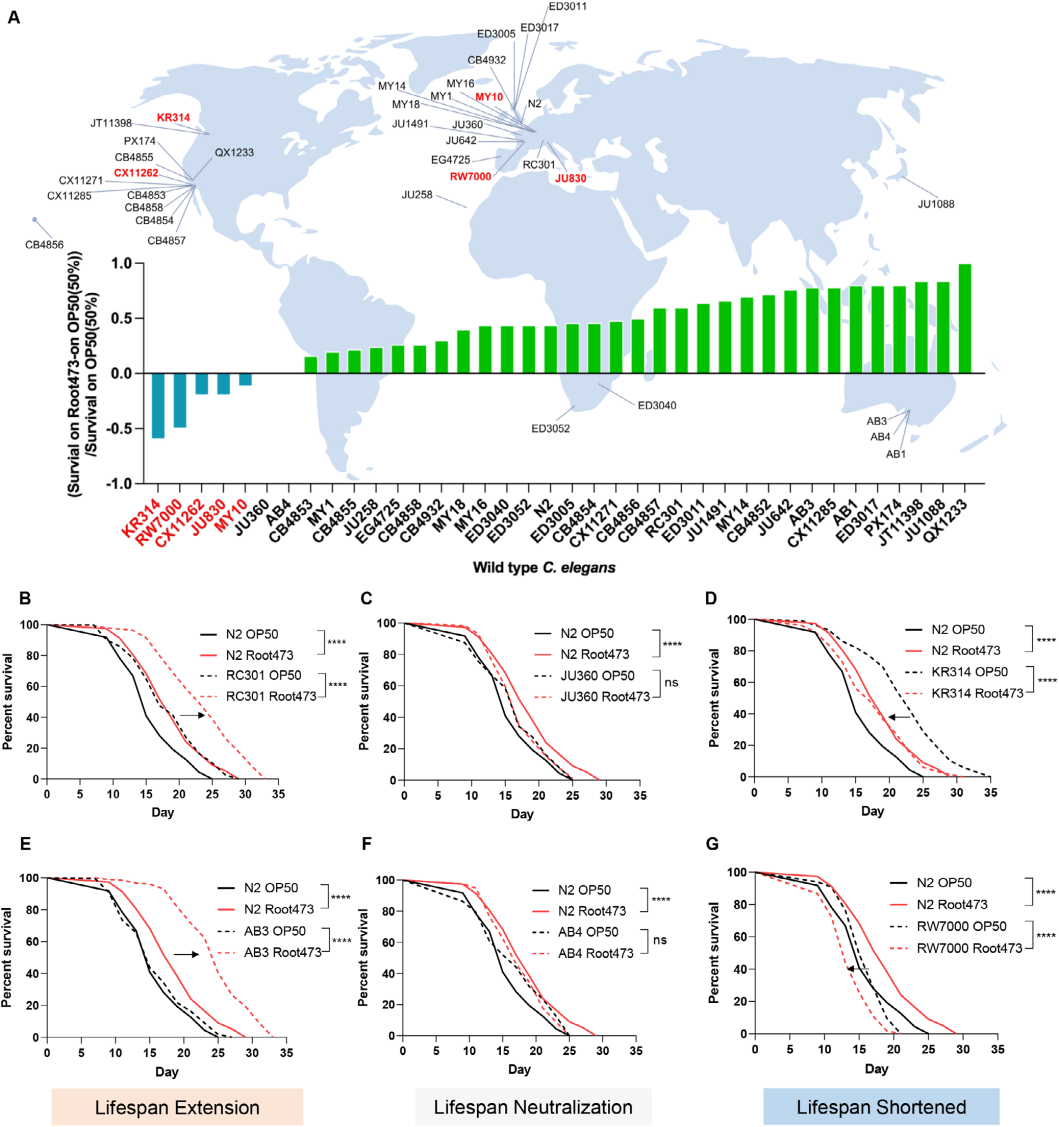
Natural genetic variation in *Caenorhabditis elegans* wild strains drives divergent lifespan responses to *Variovorax* sp. Root473. (A) Geographical distribution and lifespan variation: Map depicting the location of 38 wild‐type *C. elegans* strains (excluding CB4852) and their survival change ratio when fed on Root473 compared to OP50. Strains in green exhibit a longer lifespan on Root473, whereas strains in blue show a shorter lifespan on Root473. (B) Lifespan analysis of WT animals N2 and RC301 fed with OP50 and Root473. (C) Lifespan analysis of WT animals N2 and JU360 fed with OP50 and Root473. (D) Lifespan analysis of WT animals N2 and KR314 fed with OP50 and Root473. (E) Lifespan analysis of WT animals N2 and AB3 fed with OP50 and Root473. (F) Lifespan analysis of WT animals N2 and AB4 fed with OP50 and Root473. (G) Lifespan analysis of WT animals N2 and RW7000 fed with OP50 and Root473. ****p < 0.0001, ns denotes *p* > 0.05 via log‐rank (Mantel–Cox) test. Source data are provided in Table S10.

To determine whether natural genetic variations similarly modulate lifespan responses to the microbiota, we assessed 38 wild *C. elegans* strains, sourced globally, exposed to either standard *Escherichia coli* OP50 or *Variovorax* sp. Root473 (Zhang, Wang, et al. 2021; Yin et al. 2017; Lee et al. 2024). Consistent with our findings in genetic mutants, natural isolates exhibited a spectrum of longevity responses (Figure 2A; Table S3).

The majority of strains (31/38), such as RC301 and AB3, exhibited significantly prolonged lifespans on Root473 (Figure 2A,B,E). A second group, including JU360 and AB4, showed no significant phenotypic shift between the two bacterial diets (Figure 2A,C,F). Crucially, a distinct subset of five strains, including KR314 and RW7000, experienced significantly shortened lifespans when fed Root473 (Figure 2A,D,G; Table S3). To ensure the robustness of these findings, we performed independent secondary validation on six representative strains, which consistently reproduced the initial phenotypic groupings (Figure 2B–G; Table S10). Interestingly, these divergent outcomes did not correlate with geographic origin or broad genetic similarity, suggesting that specific, potentially rare, host genetic variants drive the differential response to Root473.

### 2.3 QTL Mapping Identifies Genetic Modifier of Microbial Longevity

**FIGURE 3:**
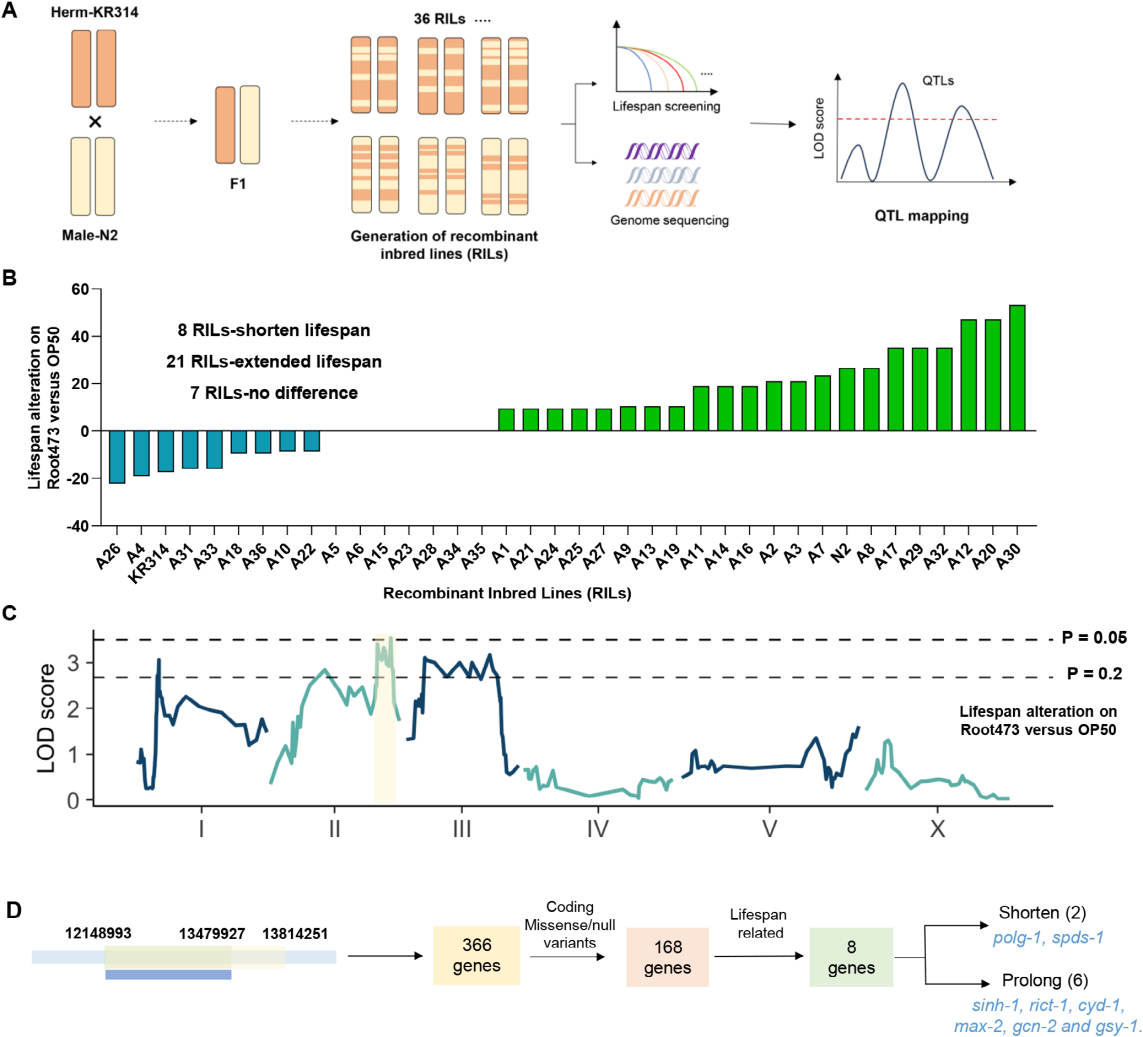
Identification of candidate genetic loci modulating bacterial‐mediated longevity through QTL analysis. (A) Recombinant inbred line (RIL) construction. Parental strains KR314 (hermaphrodites) and N2 (male) were crossed, and F1 progeny were self‐bred to produce F2 offspring. Single F2 individuals were then self‐bred across generations to establish RILs, which underwent deep sequencing and lifespan assays for quantitative trait loci (QTL) mapping to uncover genetic determinants. (B) Longevity effect of Root473 on RILs. The vertical shows the lifespan variation of RILs on Root473 relative to OP50, calculated as % change in median lifespan. (C) QTL mapping of Root473/OP50 lifespan differences. The logarithm of the odds (LOD) score is plotted across the genomic positions. Dashed lines represent suggestive (*p* < 0.2) and significant (*p* < 0.05) thresholds. (D) Gene screening within the QTL region (II: 12148993–13479927). Strategies for identifying genes within the shared QTL region potentially influencing lifespan.

To identify the genetic basis underlying these divergent microbial responses, we performed quantitative trait locus (QTL) mapping using recombinant inbred lines (RILs) derived from a cross between the Root473‐sensitive strain KR314 and the Root473‐responsive N2 (Figure 3A). We assessed the lifespan of 36 RILs and their parental strains on both *E. coli* OP50 and *Variovorax* sp. Root473 (Figure 3B; Table S4).

Our phenotypic analysis revealed several key insights: (1) Transgressive inheritance: while most RILs displayed lifespan responses intermediate to the parents, several lines exhibited phenotypes exceeding the parental range 2 shorter than KR314; 7 longer than N2 on Root473, indicating a polygenic contribution (Figure 3B). (2) Phenotypic correlation: Interestingly, the basal lifespan on OP50 was strongly negatively correlated with the lifespan alteration triggered by Root473 (*r* = −0.8451, *p* < 0.0001; Figure S1C). Specifically, RILs that recovered the pro‐longevity response to Root473 tended to have shorter basal lifespans on OP50 compared to KR314 (Figure 3B; Figure S1A). This correlation suggested a shared genetic basis for both traits.

Using the genetic diversity across the 36 RILs, we identified a major QTL on Chromosome II (positions 12148993–13814251) associated with the lifespan response to Root473 (Figure 3C; Table S5). A highly significant QTL for basal lifespan on OP50 also mapped to a nearly identical region (positions 12148993–13479927) (Figure S1B).

### 2.4 Identification and Validation of the Causal Locus via Precise Allelic Recapitulation

We focused on the overlapping QTL interval (II: 12148993–13479927), which contains 366 genes (Figure 3D). Using criteria that prioritize coding variants and known longevity‐related genes, including the presence of “Coding Missense/null variants” and “genes previously known to correlate with longevity,” we filtered this list to eight candidates (Table S6). Among these, two genes (*polg‐1* and *spds‐1*) were associated with shorter lifespans on basal OP50 (Bratic et al. 2009; Mansfeld et al. 2015), whereas the other six genes (*sinh‐1*, *rict‐1*, *cyd‐1*, *max‐2*, *gcn‐2*, and *gsy‐1*) had mutations previously reported to extend the lifespan of *C. elegans* on basal diets (Figure 3D; Table S6) (Hansen et al. 2005; Seo et al. 2018; Dottermusch et al. 2016; Derisbourg et al. 2021; Xue et al. 2007; Soukas et al. 2009).

**FIGURE 4:**
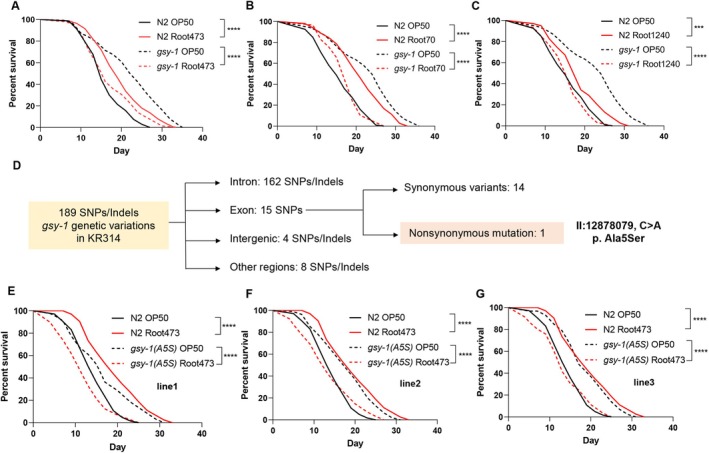
Uncovering *gsy‐1*(glycogen metabolism) as a causal genetic locus modulating microbial‐induced lifespan heterogeneity through QTL mapping and functional analysis. (A) Lifespan analysis of WT animals and *gsy‐1(gk397885)* mutants fed with OP50 and Root473. (B, C) Lifespan of *gsy‐1(gk397885)* mutants on various Root‐derived bacteria: Lifespan comparisons for *gsy‐1* mutants fed OP50 versus Root70 and Root1240. (D) Genetic variation analysis of *gsy‐1* in KR314. Flowchart showing the distribution of 189 genetic variants. Among 15 exon SNPs, only one nonsynonymous mutation (p. Ala5Ser) was found. (E–G) Lifespan analysis of WT animals and *gsy‐1(A5S)* mutants generating three lines (E, line1; F, line2; and G, line3) fed with OP50 and Root473. ****p < 0.0001, ***p < 0.001 via log‐rank (Mantel–Cox) test. Source data are provided in Table S10.

Functional validation using available *C. elegans* mutants revealed that only *gsy‐1(gk397885)*, the sole glycogen synthase gene, encodes the sole glycogen synthase in *C. elegans* , functionally analogous to human GYS1/2 (GYS1 in muscle and GYS2 in liver) (McCorvie et al. 2022; Kanungo et al. 2018), mirrored the parental KR314 phenotype: extended basal lifespan on OP50 coupled with hypersensitivity and shortened lifespan on Root473 (Figure 4A; Figure S2). This reduced lifespan phenotype was not limited to Root473; *gsy‐1* mutants also showed pronounced lifespan shortening when exposed to other *Proteobacteria* species, including Root70 and Root1240, which normally extend the lifespan of wild‐type N2 worms (Figure 4B,C).

To determine if natural genetic variation in KR314 accounted for this phenotype, we analyzed the *gsy‐1* locus via whole‐genome sequencing. Among 189 variants identified in KR314, only one nonsynonymous mutation was present: a C>A transition (II: 12878079) resulting in an Ala5Ser (A5S) substitution in the N‐terminus of the GSY‐1 protein (Figure 4D; Table S7).

To definitively test if the *gsy‐1(A5S)* variant drives the divergent lifespan response, we used CRISPR‐Cas9 genome editing to introduce this specific point mutation into the N2 background. Three independent *gsy‐1(A5S)* lines were generated. Remarkably, the A5S mutation alone was sufficient to perfectly recapitulate the complex KR314 phenotype (Figure 4E–G). These engineered animals were long‐lived on OP50 but showed significantly shortened lifespans upon exposure to Root473, matching the behavior of both the parental KR314 and the *gsy‐1* knockout. The Ala5Ser mutation resides in the N‐terminal domain, a region that interacts with many partners like GYG‐1 (Glycogenin) to regulate glycogen synthesis (McCorvie et al. 2022; Hunter et al. 2015). Collectively, these results demonstrate that natural variation in glycogen metabolism acts as a critical switch, determining whether a specific microbial exposure promotes longevity or accelerates decline.

### 2.5 Transcriptomic Profiling Reveals a Conserved “Redox Switch” Mediating Root473‐Induced Oxidative Stress

Given the shared lifespan‐shortening phenotype observed in *skn‐1*, KR314, and *gsy‐1* deficiency animals (including both the *gsy‐1(gk397885)* allele and the *gsy‐1(A5S)* knock‐in lines) upon Root473 exposure, we hypothesized that a common physiological vulnerability underlies these susceptible backgrounds. We utilized SKN‐1 (Lehrbach and Ruvkun 2019, 2016; Lehrbach et al. 2019), a widely studied regulator of stress response, as a mechanistic anchor to explore this interaction.

**FIGURE 5:**
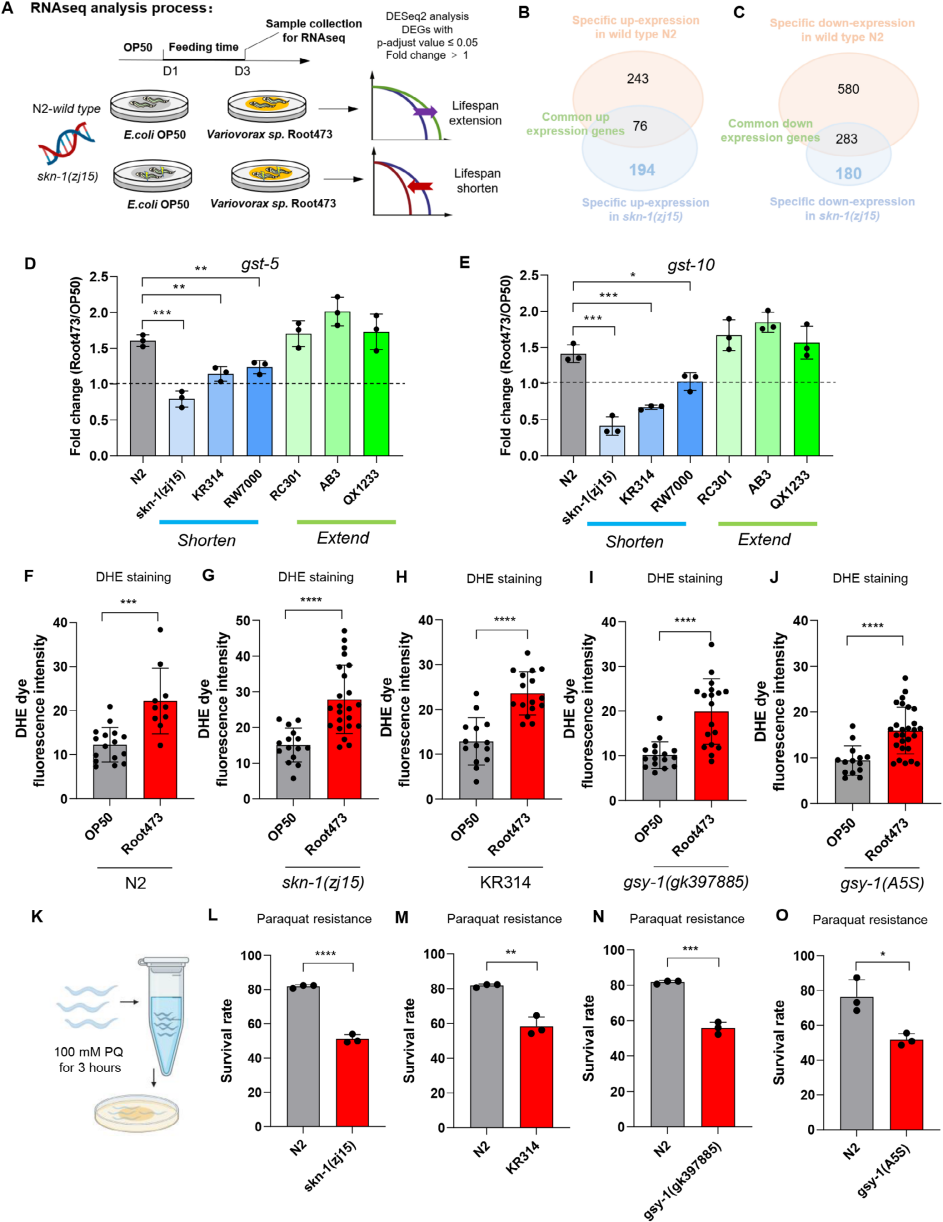
Vulnerability to Root473‐induced toxicity is driven by deficient oxidative stress resistance and divergent transcriptomics. (A) Transcriptome analysis workflow. Wild‐type N2 and *skn‐1(zj15)* mutant worms were grown on OP50 until Day 1 of adulthood, then transferred to either OP50 or Root473 for 2 days. Samples were collected on Day 3 for RNA sequencing. (B) Genetic‐context gene expression. Venn diagram shows genes uniquely upregulated in N2 (243 genes) and *skn‐1(zj15)* mutants (194 genes) on Root473, with 76 genes commonly upregulated in both. (C) Genetic‐context gene expression. Venn diagram shows genes uniquely down‐regulated in N2 (580 genes) and *skn‐1(zj15)* mutants (180 genes) on Root473, with 283 genes commonly down‐regulated in both. (D, E) Expression of glutathione metabolism genes in response to Root473: Fold changes of *gst‐5* (D) and *gst‐10* (E) in WT‐N2, *skn‐1(zj15)* mutants, KR314, RW7000, RC301, AB3, and QX1233 grown on Root473 or OP50, as analyzed by RT‐qPCR (*n* = 3 independent experiments). (F, G) ROS levels under different bacterial conditions: DHE fluorescence intensity in WT (F) and *skn‐1(zj15)* mutants (G) raised on OP50 and Root473 (*n* = 16 for OP50, *n* = 10 for Root473 in WT; *n* = 15 for OP50, *n* = 23 for Root473 in *skn‐1(zj15)*). (H) DHE dye fluorescence intensity measurement in KR314 on OP50 and Root473 diets, with sample sizes *n* = 14 (OP50) and *n* = 16 (Root473), showing relative ROS levels. (I) DHE fluorescence intensity comparison for *gsy‐1(gk397885)* mutants on OP50 and Root473, with *n* = 16 (OP50) and *n* = 18 (Root473). (J) DHE fluorescence intensity comparison for *gsy‐1(A5S)* mutants on OP50 and Root473, with *n* = 14 (OP50) and *n* = 27 (Root473). (K–O) Paraquat resistance assay. (K) Experimental schematic. (L–N) Survival rates of *skn‐1(zj15)* (L), KR314 (M), and *gsy‐1(gk397885)* mutants (N) (WT‐N2 derived from the same experimental cohort). (O) Survival rate of *gsy‐1(A5S)* mutants. (*n* = 3 independent experiments). ****p < 0.0001, ***p < 0.001, **p < 0.01, *p < 0.05, and ns denotes *p* > 0.05 via unpaired two‐tailed Student's t test. Error bars represent SEM. Source data are provided in Table S10.

To determine how host genetics modulate the impact of Root473, we performed RNA sequencing (RNA‐seq) on both wild‐type (WT) and *skn‐1* mutant animals (Figure 5A–C). Root473 exposure in wild‐type worms significantly upregulated glutathione metabolism genes (e.g., *gst‐5*, *gst‐10*, *gst‐36*, and *gpx‐5*), which are canonical SKN‐1 targets essential for ROS neutralization (Figure 5B; Figure S3A) (Sirakawin et al. 2024; Gusarov et al. 2021). Although both genotypes induced certain oxidoreductases (e.g., Cytochrome P450s) (Burns et al. 2023), *skn‐1* mutants specifically upregulated ferroptosis‐related markers (e.g., *ftn‐1*, *ftn‐2*), suggesting a shift from productive stress signaling to iron‐mediated oxidative damage (Romero‐Afrima et al. 2020; Kim et al. 2022) (Figure 5B; Figure S3B,C).

We hypothesized that this failure to activate glutathione‐mediated defense might be a universal signature of microbial susceptibility. Indeed, qPCR analysis of additional long‐lived (RC301, AB3, and QX1233) versus short‐lived (KR314, RW7000) strains confirmed that glutathione activation is a conserved prerequisite for Root473‐mediated longevity (Figure 5D,E). Interestingly, we also observed a reciprocal pattern in methionine metabolism, which was down‐regulated in long‐lived strains but not in susceptible ones (Figure S3D,E). This signature mirrors established longevity models like methionine restriction, suggesting that Root473 promotes lifespan via a specific metabolic reprogramming (Parkhitko et al. 2019).

Importantly, we found that alternative pathways, including the detoxification system (Figure S4A–H) (e.g., CYPs [*cyp‐25A2*, *cyp‐33C7*, *cyp‐33E1*, and *cyp‐33E2*]; Larigot et al. 2022; Lim et al. 2025) and UGTs (*ugt‐22*, *ugt‐23*, *ugt‐49*, and *ugt‐61*) (Asif et al. 2024; Fontaine and Choe 2018) and innate immune‐related genes (Figure S4I–L) (*lys‐2*, *clec‐4*, *clec‐67*, and *clec‐85*) (Tse‐Kang et al. 2025; Fletcher et al. 2019)—were changed similarly across all genetic backgrounds regardless of their lifespan outcomes. These results indicate that the oxidative stress response, rather than generalized immune or detoxification pathways, is the core determinant of host‐microbe longevity heterogeneity.

We next evaluated whether Root473 acts as a source of oxidative stress. Using DHE staining, we found that ROS levels were elevated in all strains—including N2 upon Root 473 exposure (Figure 5F–J) (Gusarov et al. 2021). Thus, ROS elevation appears to be a general effect of Root473 rather than a specific trait of short‐lived strains. To assess oxidative defense integrity, we challenged the animals with paraquat (Figure 5K). Notably, only *skn‐1*, KR314, and *gsy‐1* mutants exhibited hypersensitivity to the external ROS inducer, confirming a common defect in oxidative buffering capacity (Figure 5L–O).

### 2.6 RAS Pathway Inhibition Rescues Susceptibility to Root473

**FIGURE 6:**
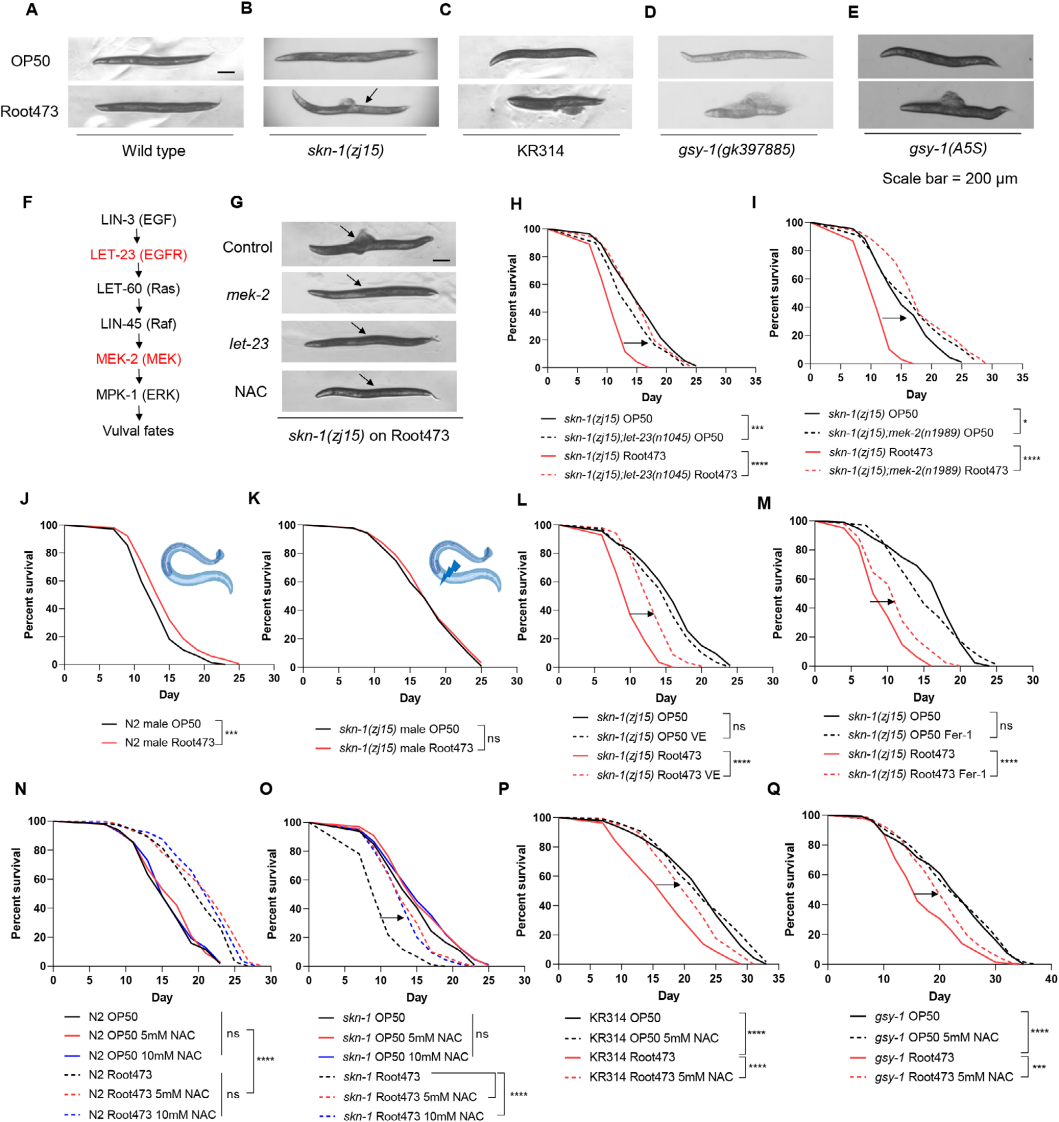
Suppression of RAS/RAF signaling or antioxidant supplementation reverses microbiota‐driven physiological collapse and lifespan. (A) Body integrity of WT animals: Representative images of body integrity in WT animals fed on OP50 and Root473 during aging. Scale bar: 200 μm. (B) Body integrity of *skn‐1(zj15)* mutants: Representative images of body integrity in *skn‐1* animals fed on OP50 and Root473 during aging. (C) Body integrity of KR314 animals: Representative images of body integrity in KR314 animals fed on OP50 and Root473 during aging. (D) Body integrity of *gsy‐1(gk397885)* mutants: Representative images of body integrity in *gsy‐1(gk397885)* animals fed on OP50 and Root473 during aging. (E) Body integrity of *gsy‐1(A5S)* mutants: Representative images of body integrity in *gsy‐1(A5S)* animals fed on OP50 and Root473 during aging. (F) RAS–RAF–ERK pathway in *Caenorhabditis elegans*. Diagram of the signaling pathway, which regulates vulva morphogenesis. (G) Body integrity of *skn‐1(zj15)* mutants with RAS pathway mutations (*let‐23*, *mek‐2*) or NAC supplementation fed on Root473 during aging. Scale bar: 200 μm. (H) Lifespan analysis of *skn‐1* mutants and *skn‐1(zj15); let‐23(n1045)* mutants fed with OP50 and Root473. (I) Lifespan analysis of *skn‐1* mutants and *skn‐1(zj15); mek‐2(n1989)* mutants fed with OP50 and Root473. (J) Lifespan analysis of N2 males fed with OP50 and Root473. (K) Lifespan analysis of *skn‐1(zj15)* males fed with OP50 and Root473. (L) Lifespan analysis of *skn‐1(zj15)* mutants fed on OP50 and Root473 with supplementation of Vitamin E (2.5 mM). (M) Lifespan analysis of *skn‐1(zj15)* mutants fed on OP50 and Root473 with supplementation of ferrostatin‐1 (100 μM). (N) Lifespan analysis of WT animals on OP50 and Root473 with different concentrations of NAC (5 and 10 mM). (O) Lifespan analysis of *skn‐1(zj15)* mutants on OP50 and Root473 with different concentrations of NAC (5 and 10 mM). (P) Lifespan analysis of KR314 fed on OP50 and Root473 with NAC supplementation (5 mM). (Q) Lifespan analysis of *gsy‐1(gk397885)* mutants fed on OP50 and Root473 with NAC supplementation (5 mM). ****p < 0.0001, ***p < 0.001, *p < 0.05, ns denotes *p* > 0.05 via log‐rank (Mantel–Cox) test. Source data are provided in Table S10.

Additionally, we observed that the accelerated aging in susceptible hosts (*skn‐1*, KR314, and *gsy‐1*) was frequently accompanied by severe vulval tissue damage (Figure 6B–E; Figure S5B–E), a phenotype almost absent in wild‐type worms (Figure 6A; Figure S5A). Given that the EGF‐RAS‐MAPK signaling cascade is the primary driver of vulval morphogenesis (Han et al. 1993; Zand et al. 2011; Shin and Reiner 2018; Branicky et al. 2022), we hypothesized that the pathological effects of Root473 might be mediated through the ectopic activation or sensitivity of this pathway under oxidative stress. To test this, we utilized loss‐of‐function mutations in key pathway components, including *let‐23* (EGFR) and *mek‐2* (MEK). Remarkably, suppressing EGF‐RAS‐MAPK signaling significantly mitigated the vulval defects and restored the lifespan of *skn‐1* mutants on Root473 (Figure 6F–I; Figure S5F,G). Furthermore, male *skn‐1* worms—which lack vulval structures—were protected from the early‐death phenotype observed in hermaphrodites (Figure 6J,K). These results identify the vulva as a focal point of tissue‐specific susceptibility to microbial‐induced oxidative damage.

### 2.7 Targeted Antioxidants Reverse Microbiota‐Driven Accelerated Aging

Because the damage in susceptible hosts appeared linked to redox imbalance, we explored the therapeutic potential of lipid‐targeted and general antioxidants. Supplementation with ferrostatin‐1 (Fer‐1) or Vitamin E (Qin et al. 2022), which specifically inhibit lipid peroxidation, partially rescued the lifespan of *skn‐1* mutants on Root473 (Figure 6L,M). The most profound effects were observed with N‐acetylcysteine (NAC) treatment. Although NAC did not further extend the already long lifespan of wild‐type animals on Root473, it provided a near‐complete rescue of the pathological phenotypes in *skn‐1* mutants (Figure 6N,O). Specifically, NAC supplementation restored body integrity, preserved the intestinal barrier, maintained mitochondrial morphology, and reversed the shortened lifespan (Figure 6G,O; Figure S6). Importantly, this pharmacological rescue was not limited to *skn‐1* mutants; NAC also significantly restored the lifespans of KR314 and *gsy‐1* mutants exposed to Root473 (Figure 6P,Q). Together, these findings demonstrate that the outcomes of host‐microbiota interactions are dictated by the interplay between oxidative stress and tissue‐specific sensitivity. Our results suggest that detrimental aging effects driven by the microbiome can be effectively reversed by interventions that reinforce redox balance, either through the modulation of genetic signaling pathways or direct antioxidant supplementation.

## 3 Discussion

**FIGURE 7:**
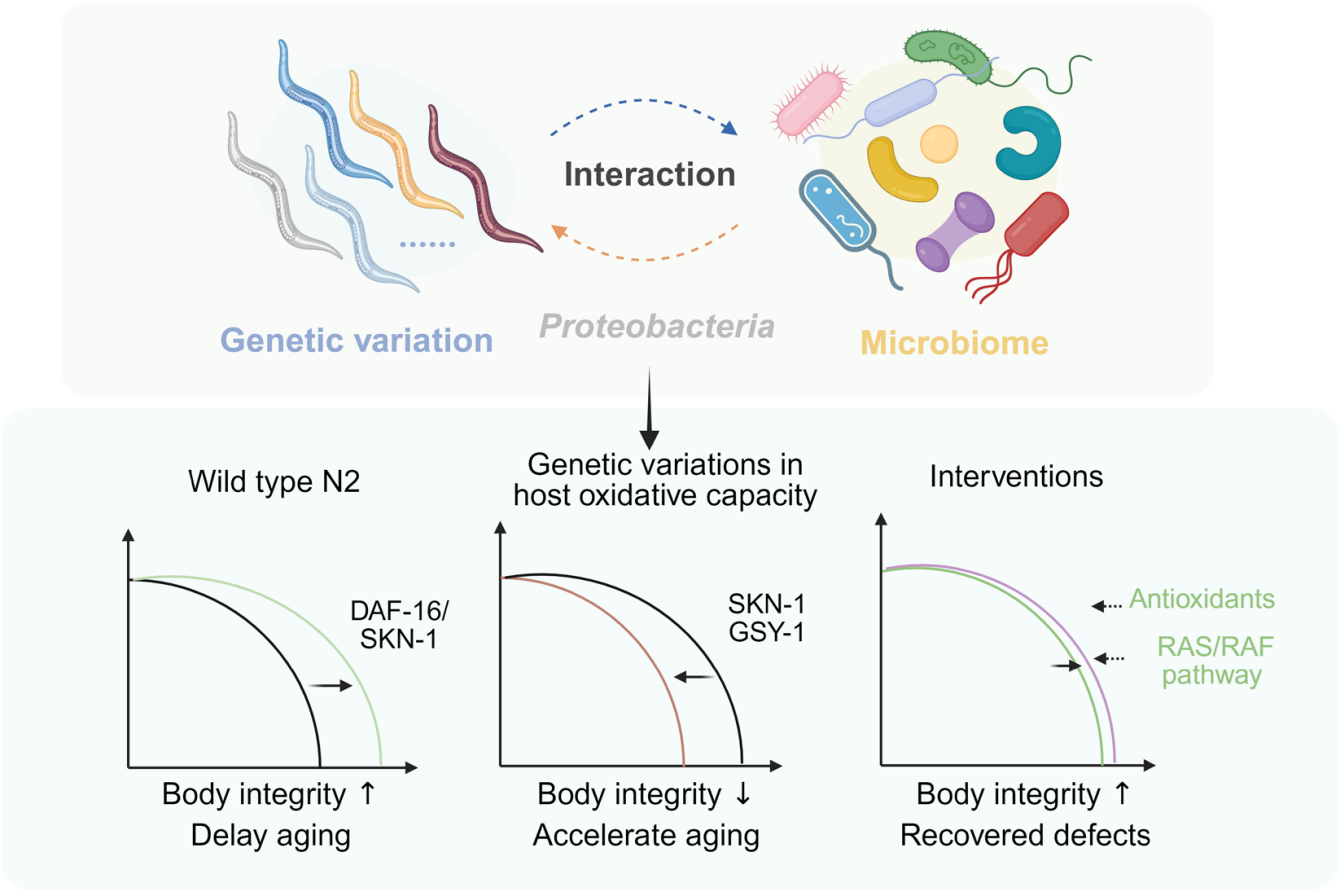
Schematic model illustrating how host genetic variation shapes lifespan response to microbial interventions. This model depicts the heterogeneous regulation of lifespan mediated by bacterial species, exemplified by *Variovorax* sp. Root473. Longevity signal (robust hosts): In wild‐type *Caenorhabditis elegans* (N2), Root473 acts as a beneficial stimulus that requires SKN‐1 and DAF‐16 to promote redox balance, maintain tissue integrity, and extend lifespan. Toxicity trigger (susceptible hosts): In hosts with genetic variations affecting oxidative stress resilience (e.g., mutations or natural variants in SKN‐1 and GSY‐1), Root473 compromises body integrity, reduces lifespan, and exacerbates the age‐related deterioration. Therapeutic reversal: The detrimental effects of the microbiome in susceptible backgrounds are reversible. Both pharmacological interventions (antioxidants: NAC, Vitamin E, Fer‐1) and genetic modulation of the RAS pathway can significantly counteract the detrimental effects of Root473. Created with BioRender.

Aging is a multifaceted biological process shaped by both intrinsic and extrinsic factors. Although much is known about canonical genetic pathways and environmental cues that influence aging, our understanding of how natural genetic variations interact with microbial influences remains incomplete. In this study, we used *C. elegans* as a model to systemically investigate how host genetic diversity modulates lifespan outcomes in response to microbial interventions. Our findings reveal striking heterogeneity in lifespan regulation across genetically distinct *C. elegans* strains, with oxidative stress resilience emerging as a central determinant of this variability. These results underscore the importance of incorporating host genetics into aging research and suggest the potential for personalized, microbial‐based therapies to promote healthy aging (Figure 7).

Our work reinforces the growing consensus that effective anti‐aging interventions must take into account individual variation, including genetic background, sex, and age. Previous studies have demonstrated sex‐specific responses to lifespan‐extending interventions. For example, rapamycin predominantly extends lifespan in female flies (Regan et al. 2022), whereas acarbose (ACA) and 17‐α estradiol (17aE2) are more effective in male mice, particularly regarding improvements in insulin sensitivity and glucose metabolism (Garratt et al. 2017). Similarly, the efficacy of certain interventions, such as metformin, depends on the timing of administration: beneficial when applied early, but detrimental if introduced later in life due to metabolic collapse (Espada et al. 2020). Additionally, host genetic background can significantly influence responses to dietary restriction (DR) intervention, as shown in female diversity outbred (DO) mice (Di Francesco et al. 2024). However, the specific genetic variations responsible for this variability have remained largely undefined.

Our findings now provide a framework to dissect such genetic contributions. By identifying *skn‐1* and *gsy‐1* as genetic modulators of lifespan responses to specific bacterial strains, we demonstrate how microbial interventions can produce opposing outcomes, either extending or shortening lifespan, depending on the host's genetic makeup. These results underscore the importance of incorporating host genotype into the design and evaluation of aging interventions, especially those involving microbiota.

Oxidative stress emerged as a key mechanistic axis underlying differential lifespan responses. Reactive oxygen species (ROS) are essential signaling molecules, but their accumulation beyond buffering capacity contributes to cellular damage and aging (Lennicke and Cochemé 2021; Bazopoulou et al. 2019). Our study reveals that mutations in *gsy‐1*, encoding the sole glycogen synthase in *C. elegans* , result in heightened sensitivity to microbial‐induced oxidative stress. Glycogen has been proposed to buffer oxidative damage by supporting NADPH and GSH regeneration (Gusarov et al. 2017). Accordingly, *gsy‐1* mutants exhibit redox imbalance and accelerated aging when exposed to *Proteobacteria* such as *Variovorax* sp. Root473, a phenotype that can be rescued by antioxidant supplementation (e.g., NAC). These observations align with prior findings that mutations in *alh‐6*, which affect proline metabolism, also increase ROS and lead to diet‐specific lifespan reduction (Pang and Curran 2014). Together, these findings suggest that oxidative stress resilience is a fundamental determinant of how host organisms respond to microbial cues during aging.

Our QTL mapping analysis further highlights the genetic complexity underlying host‐microbiota interactions. Lifespan assays in recombinant inbred lines (RILs) revealed non‐additive and transgressive segregation patterns, indicating that nonlinear genetic interactions govern the effects of microbial interventions on aging. These findings reinforce the concept that microbial modulation of host physiology cannot be generalized across genotypes and must be evaluated in a context‐specific manner.

These insights have important implications beyond *C. elegans* . With growing interest in microbial‐based therapies, it is becoming increasingly clear that probiotics and engineered microbial consortia may have variable effects across individuals. For instance, the commonly used *Lactobacillus* genus can have both beneficial and detrimental effects depending on context. It has been shown to produce indoles from tryptophan metabolism, activating the aryl hydrocarbon receptor (AhR) and potentially promoting tumor growth and immune suppression in pancreatic ductal adenocarcinoma (PDAC) (Hezaveh et al. 2022). Our findings add another layer to this complexity by showing that even a single microbial species can have dichotomous effects, extending or reducing lifespan, based solely on host genetic variation.

Taken together, our results advocate for a precision approach to microbiome intervention design, guided by host genetics and physiological state. The variability we observed in oxidative stress responses, mitochondrial morphology, and tissue integrity further emphasizes that the same microbial signal may lead to adaptive or maladaptive outcomes depending on the host's capacity for stress resilience.

### 3.1 Limitations of the Study

Our study identifies a critical genetic axis governing host‐microbe longevity, yet several limitations remain. Due to the intensive nature of longitudinal lifespan phenotyping, our RILs mapping population was powered to detect major‐effect loci but likely lacks the resolution to identify other‐effect modifiers or complex epistatic interactions. Furthermore, while CRISPR‐Cas9 allelic recapitulation definitively proves that the *gsy‐1(A5S)* variant is a primary driver of the Root473‐sensitive phenotype, it may not account for the total phenotypic variance in the KR314 background. Finally, the precise biochemical mechanism by which glycogen metabolism coordinates with the *skn‐1*‐mediated antioxidant response remains to be fully elucidated. Future research utilizing integrated metabolomics will be essential to map the complete molecular bridge between host metabolic state and microbial‐driven redox homeostasis.

## 4 Methods

### 4.1 Bacterial Resources and Culturing

*Escherichia coli* OP50 was used as the control bacterial strain in this study. *Arabidopsis* root‐associated bacterial isolates were kindly provided by Dr. Yang Bai's lab at Peking University, Beijing, China (Bai et al. 2015). Detailed information on the bacterial communities is available in Table S1.

For culturing bacteria in liquid medium, individual colonies were grown to logarithmic phase (OD_600_ = 0.8–1.0) at 30°C in trypticase soy broth (TSB). The bacteria were then harvested by centrifugation at 4000 *g* for 10 min, washed twice with TSB buffer, and re‐suspended in TSB at a 5‐fold concentration.

For the preparation of lifespan plates and other physiology assays, 100–150 μL of the concentrated bacterial suspension was inoculated onto each 60 mm NGM plate. For experiments using dead bacteria feeding, the 5‐fold concentrated suspension was inactivated by heat treatment at 60°C for 40 min. The treated bacterial suspension was then seeded onto the NGM plates supplemented with kanamycin (50 mg/mL) to prevent regrowth.

### 4.2 *Caenorhabditis elegans* Strains and Maintenance

*Caenorhabditis elegans* strains were maintained on standard nematode growth medium (NGM) agar plates seeded with *E. coli* OP50 at 20°C unless otherwise noted. Detailed information on all strains used in this study is provided in Table S9.

### 4.3 Intestine Permeability Assay

Intestinal barrier integrity was assessed as previously described (Qu et al. 2018). Adulthood *C. elegans* raised on either OP50 or Root473 were rinsed from NGM plates with M9 buffer. Worms were then incubated with 5% (wt/vol) erioglaucine disodium blue dye for 3 h. Following staining, animals were washed 5 times with M9 buffer to remove excess dye. Dye distribution in the worms was examined under a microscope to assess intestinal barrier integrity. In healthy animals, their dye was primarily confined to the intestinal lumen. The degree of intestinal leakage was scored on a scale from I to IV, representing intact to severely compromised barrier integrity. Each experiment was repeated at least three times.

### 4.4 Microscopy and Image Analysis

To assess body integrity, worms were anesthetized with 50 mM NaN_3_ prior to imaging. Body images were captured using a Leica M165 FC dissecting microscope. Mitochondrial morphology was visualized using a Leica‐TCS SP8 laser scanning confocal microscope. All images presented in our figures are representative of findings observed in more than five images.

### 4.5 Paraquat Resistant Assay

Synchronized Day 1 adult worms were collected and washed twice with M9 buffer. The worms were then incubated in M9 containing 100 mM paraquat for 3 h at 20°C, followed by an overnight recovery period. Survival rates were analyzed after recovery, and the assay was repeated in three independent biological replicates.

### 4.6 DHE Staining Assay

The fluorescent dye dihydroethidium (DHE, Invitrogen) was used to quantify ROS production. A 500 μM stock solution of DHE was prepared in DMSO. Synchronized Day 1 adult worms fed on OP50 or Root473 were collected, washed twice with M9 buffer, and stained in M9 containing 5 μM DHE for 30 min at 20°C. After staining, worms were extensively washed with M9, and images were captured immediately using a Leica‐TCS SP8 laser scanning confocal microscope. Fluorescence intensity was quantified using the ImageJ software.

### 4.7 RNA Extraction and RT‐qPCR

Synchronized Day 1 adult worms, cultivated on the OP50 diet, were transferred to OP50 or Root473 and allowed to feed for 48 h. The Day 3 worms were then collected using M9 buffer. To each sample, 1 mL TRIzol (Invitrogen) was added, and samples were homogenized by repeated freeze–thaw cycles in liquid nitrogen 5–6 times. Total RNA was extracted with chloroform, followed by ethanol washing and isopropanol precipitation. Complementary DNA (cDNA) was synthesized using the ReverTra Ace qPCR RT Master Mix with gDNA remover (Toyobo Cat. no. FSQ‐301). RT‐qPCR was performed with SYBR Green Realtime PCR Master Mix (Toyobo Cat. no. QPK‐201) on a Bio‐Rad CFX 96 Real‐Time PCR Detection System. Gene expression was normalized to *act‐3* mRNA levels, and fold changes were calculated using the comparative $2^{-∆∆C_{t}}$ method. Each assay was conducted in three independent biological replicates. And the primers used for qPCR analysis were shown in Table S11.

### 4.8 RNA‐Seq Analysis

Synchronized Day 1 adult worms were initially cultivated on NGM plates with OP50 at 20°C, and then were transferred to plates seeded with either OP50 or Root473 plates. After 48 h, Day 3 worms from each condition were collected by washing with M9 buffer, followed by the addition of 1 mL TRIzol reagent (Invitrogen) and snap‐freezing at −80°C. Each treatment condition included 3 biological replicates. RNA quality was assessed using an Agilent 5300 and NanoDrop 2000, and samples with high RNA integrity were selected for library construction and RNA sequencing, which was performed by MajorBio company.

For data processing, the Fastx_toolkit (Version 0.0.14), Sickle, SeqPrep, and fastp (Version 0.19.5) were used for the quality control. The reference genome (Wbcel235, Bioproject: PRJNA13758) was obtained from NABI. Alignment to the worm genome was performed using Bowtie2 (Version 2.4.1), hisat2 (Version 2.1.0), TopHat (Version v2.1.1), and STAR (Version 2.7.1a). Differential expression analysis was conducted using DESeq2, with genes showing an adjusted *p*‐value ≤ 0.05 and a fold change > 1 considered as significantly differentially expressed genes (DEGs). Functional enrichment analysis of DEGs was performed using KEGG or GO annotations to identify significantly enriched pathways, which are presented in Table S8.

### 4.9 CRISPR/Cas9‐Mediated Gene Editing

The *gsy‐1(A5S)* point mutation was generated in the N2 background using CRISPR/Cas9 genome editing as previously described (Waaijers et al. 2013), with minor modifications. Briefly, three single‐guide RNAs (sgRNAs) targeting sequence (sgRNA1: 5′‐CGGCATCACTGGGAGTCAGG‐3′, sgRNA2: 5′‐GATTTAATGACGGCATCACT‐3′, and sgRNA3: 5′‐GATTTCTGGGCATTCGTGCG‐3′) of *gsy‐1* were cloned into a truncated pDD162 vector lacking Cas9 expression. A repair template containing the mutation of interest (c.G13T; p.A5S; corresponding to C>A on the reverse strand) and additional silent mutations (such as c.C9T) was designed to prevent Cas9 re‐cleavage of the edited locus. The injection cocktail comprised the Cas9 expression plasmid (40 ng/μL), the sgRNA plasmids (40 ng/μL each), the KI donor template (40 ng/μL), and a co‐injection marker *myo‐2p::tdTomato* (10 ng/μL). Following injection into N2 hermaphrodites, tdTomato‐positive F1 progeny were isolated. The *gsy‐1(A5S)* mutation was confirmed in the F2 generation through PCR amplification and sequencing.

### 4.10 Chemical Supplementation of Culture Plates

For chemical supplementation, stock solutions were prepared by dissolving each chemical in either water, DMSO, or ethanol. A 1 M stock solution of N‐Acetyl‐cysteine (NAC) was prepared in ddH_2_O, whereas a 50 mM stock solution of Fer‐1 was dissolved in DMSO, and Vitamin E was dissolved in ethanol. All stock solutions were stored at −20°C. Prior to each experiment, these stock solutions were diluted to the required final concentration directly in NGM before the plates were poured.

### 4.11 Lifespan Assay

Lifespan analysis was conducted on NGM plates at 20°C following established protocol (Dillin et al. 2002). The worms were synchronized by egg bleaching and raised on OP50, Root473, or other root‐derived bacterial isolates. Worms were monitored for viability every other day from Day 1 of adulthood. To avoid progeny interference and environmental contamination, animals were transferred to fresh NGM plates with food every second day. For experiments requiring a change in bacterial diet, worms were washed at least twice in M9 buffer containing 50 mg/mL kanamycin before being placed on the new plates. Lifespan data reflect pooled results across multiple experiments. Statistical analysis was performed using Prism9 software, and lifespan differences were determined by the log‐rank (Mantel–Cox) method for significance.

### 4.12 Construction of Recombinant Inbred Lines

To construct recombinant inbred lines, KR314 hermaphrodites were crossed with N2 males, which were induced by heat stress (4–6 h at 30°C). F1 offspring were generated by cross‐fertilization of parental lines, after which 5–6 F1 animals were randomly selected and transferred onto fresh NGM plates. After 3 days, the F2 generation was obtained through self‐fertilization of F1 worms. Individual F2 worms were isolated and inbred through single‐worm transfers for 5–6 successive generations to maximize genetic diversity and produce homozygous lines. This process ultimately yielded 36 recombinant inbred lines, which were then used for subsequent studies and whole‐genome sequencing.

### 4.13 Whole‐Genome Sequencing and SNP Calling

*Caenorhabditis elegans* strains were sequenced at an average depth of ~30× using the DNBSEQ platform with 150‐base‐pair paired‐end reads. Sequence reads were aligned to the reference genome using BWA‐MEM (Li 2013). Short variants, including SNPs and indels, were called using GATK HaplotypeCaller v4.0.5.1 (McKenna et al. 2010). Filtering criteria for high‐quality variant calls were applied as follows: for SNPs, “QD < 2.0 || FS > 60.0 || MQRankSum < −12.5 || ReadPosRankSum < −8.0 || MQ < 40.0 || SOR > 3.0”; and for indels, “QD < 2.0 || FS > 200.0 || MQRankSum < −12.5 || ReadPosRankSum < −8.0 || SOR > 10.0.” Additionally, filtering steps included retaining only biallelic polymorphisms and removing variants with a missing data rate > 20%.

### 4.14 Quantitative Trait Locus (QTL) Mapping

A RIL population consisting of 36 progenies derived from the KR314× N2 cross was used for QTL analysis. Variants with missing data or identical genotypes between the two parental strains were removed. Bin markers were constructed using the “crosspoints” function in snpbinner, with a minimum recombination exchange rate of 0.01 and a minimum bin size of 5000 bp. Bin files from individual chromosomes were reformatted and merged, resulting in 532 bin markers. Genetic maps were estimated with est.map in qtl2, and Spearman correlations between genetic and physical distances were calculated for each linkage group. QTL mapping was performed using the qtl2 R package to associate phenotypes with these bin markers (Broman et al. 2019). Whole‐genome QTL scans were conducted using the Haley–Knott regression method, with significance thresholds determined by 1000 permutation tests. Significant QTLs were identified using the peak function in qtl2 with a threshold of *p* < 0.05 and a drop of 0.5.

### 4.15 Statistical Analysis

All experiments in this study were conducted independently at least two or three times, except for primary lifespan screening assays (Figures 1B, 2A, 3B), where results were consistently reproducible across biological repeats. Appropriate statistical tests were applied to figures, ensuring that data met the necessary assumptions for the chosen analyses. Statistical details including sample size (*n*), descriptive statistics (mean ± SEM), and significance levels, are provided in the related figure or figure legends. An unpaired, two‐tailed student's *t*‐test was used to compare two normally distributed groups, whereas lifespan assays were analyzed using the Mantel–Cox log‐rank test. And more details were shown in the source data Table S10.

## Author Contributions

Y.T. and X.H. conceived the study and designed the experiments. X.H. performed the lifespan screenings and conducted the lifespan experiments. X.H. performed various physiological assays such as intestinal barrier evaluation, DHE staining, paraquat resistance, and body integrity imaging. X.H. conducted the RNA‐seq analysis, qPCR experiments and *Caenorhabditis elegans* crosses. X.H. and R.Y. performed the mitochondrial morphology images. Y.G. and G.C. provided the sequencing analysis and recommendations. G.C. carried out plasmid construction and created transgenic worm strains. X.H. constructed the recombinant inbred lines, and Y.G. and F.L. helped with the bioinformatic QTL mapping analysis. Y.B. isolated the bacterial isolates, and L.L. maintained the isolates collection in our lab. X.H. prepared the original figures and source data. Y.T. and X.H. wrote the manuscript.

## Funding

This study was supported by the National Key Research and Development Program of China (2022YFA1303000), National Natural Science Foundation of China (32225025, 32321004, 32430025, 32225038), CAS Project for Young Scientists in Basic Research (YSBR‐076), and New Cornerstone Science Foundation.

## Conflicts of Interest

The authors declare no conflicts of interest.

## Supporting information


**Table S1:** List of *Arabidopsis* root‐derived bacterial collections.
**Table S2:** Medium survival changes of eight represent bacteria isolates on different genetic hosts.
**Table S3:** Lifespan analysis of *Caenorhabditis elegans* wild strains on Root473 versus OP50.
**Table S4:** Lifespan alteration on Root473 versus OP50 and lifespan alteration on OP50 of RILs.
**Table S5:** QTL mapping data including POS and LOD score.
**Table S6:** List of genes from QTL mapping region (II: 12148993–13479927).
**Table S7:** Information about SNPs/indels within *gsy‐1* in KR314.
**Table S8:** RNA‐seq analysis of different regulated genes of N2 and *skn‐1(zj15)* on Root473 versus OP50.
**Table S9:** *Caenorhabditis elegans* strains used in this study.
**Table S10:** Source data files.
**Table S11:** List of primers used for RT‐qPCR.
**Figure S1:** QTL mapping of the lifespan alteration of RILs (on OP50) traits.
**Figure S2:** Lifespan validation of candidate genes in the QTL region (II: 12148993–13479927).
**Figure S3:** Host oxidative stress capacity determines the lifespan effects of *Variovorax* sp. Root473 on *Caenorhabditis elegans* .
**Figure S4:** Detoxification pathways and innate immunity do not drive the genotype‐specific lifespan responses to Root473.
**Figure S5:** Vulval integrity and impact of RAS/RAF signaling on host lifespan.
**Figure S6:** NAC supplementation restores the mitochondrial defects and intestine integrity in *skn‐1(zj15)* mutants upon Root473 exposure.

## Data Availability

All data supporting the findings of this study are available within the paper and its Tables S1–S11 or information files. Source data are also provided for further reference. The raw sequence data for RNA‐seq generated in this study have been deposited in the Genome Sequence Archive in the National Genomics Data Center with accession number CRA021524.
