# Supplementary figures and images for: Host Oxidative Response Capacity Determines Longevity Outcomes of Microbial Interventions
Source: Aging Cell. 2026 Feb 12;25(2):e70418. doi: 10.1111/acel.70418 (PMC12901668; doi:10.1111/acel.70418)

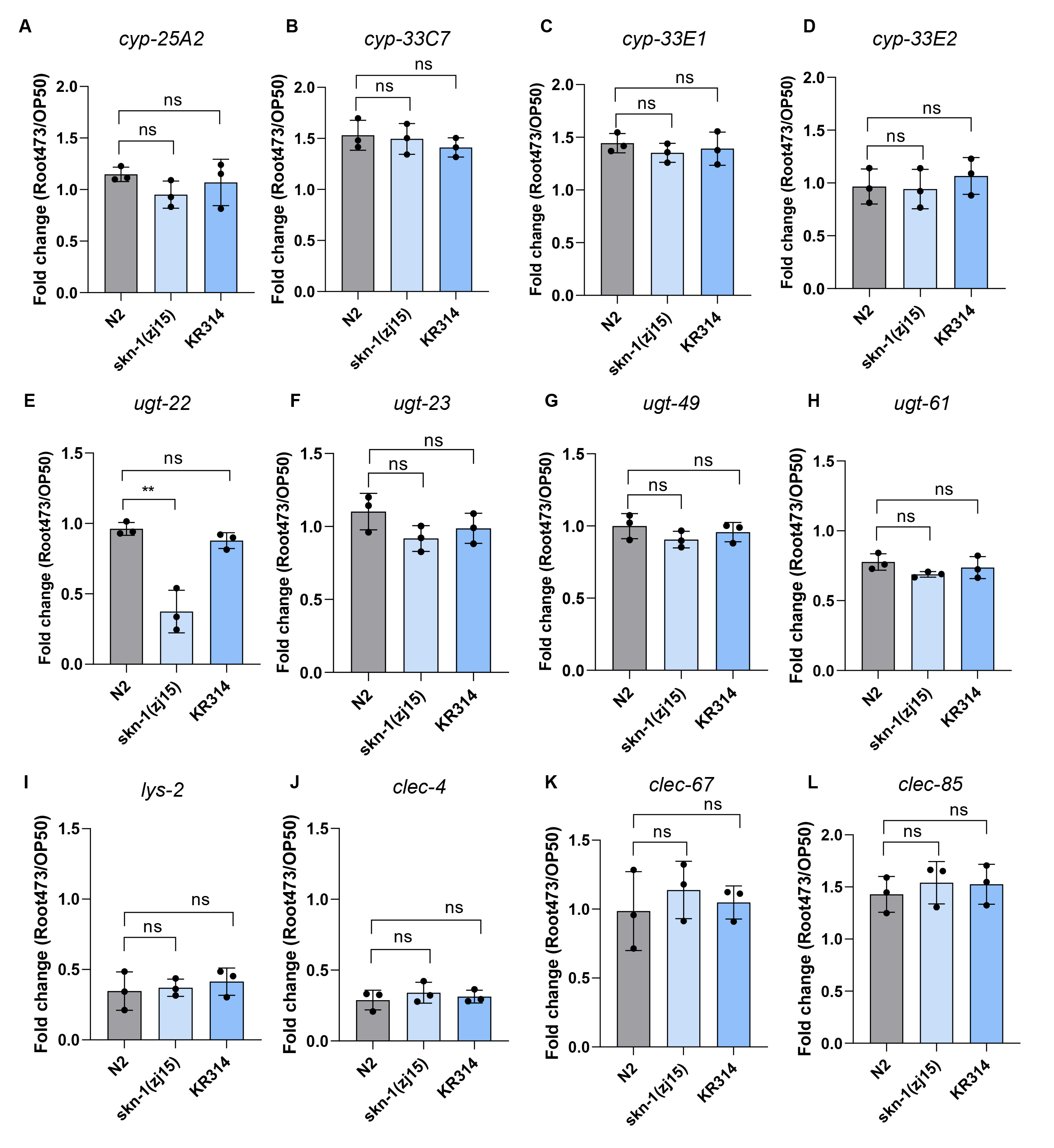

Supplement: Supplementary file 1 — Table S1: List of Arabidopsis root‐derived bacterial collections. Table S2: Medium survival changes of eight represent bacteria isolates on different genetic hosts. Table S3: Lifespan analysis of Caenorhabditis elegans wild strains on Root473 versus OP50. Table S4: Lifespan alteration on Root473 versus OP50 and lifespan alteration on OP50 of RILs. Table S5: QTL mapping data including POS and LOD score. Table S6: List of genes from QTL mapping region (II: 12148993–13479927). Table S7: Information about SNPs/indels within gsy‐1 in KR314. Table S8: RNA‐seq analysis of different regulated genes of N2 and skn‐1(zj15) on Root473 versus OP50. Table S9: Caenorhabditis elegans strains used in this study. Table S10: Source data files. Table S11: List of primers used for RT‐qPCR. Figure S1: QTL mapping of the lifespan alteration of RILs (on OP50) traits. Figure S2: Lifespan validation of candidate genes in the QTL region (II: 12148993–13479927). Figure S3: Host oxidative stress capacity determines the lifespan effects of Variovorax sp. Root473 on Caenorhabditis elegans . Figure S4: Detoxification pathways and innate immunity do not drive the genotype‐specific lifespan responses to Root473. Figure S5: Vulval integrity and impact of RAS/RAF signaling on host lifespan. Figure S6: NAC supplementation restores the mitochondrial defects and intestine integrity in skn‐1(zj15) mutants upon Root473 exposure. [file ACEL-25-e70418-s001.zip › 1_Supplementary Figure-4.tif]

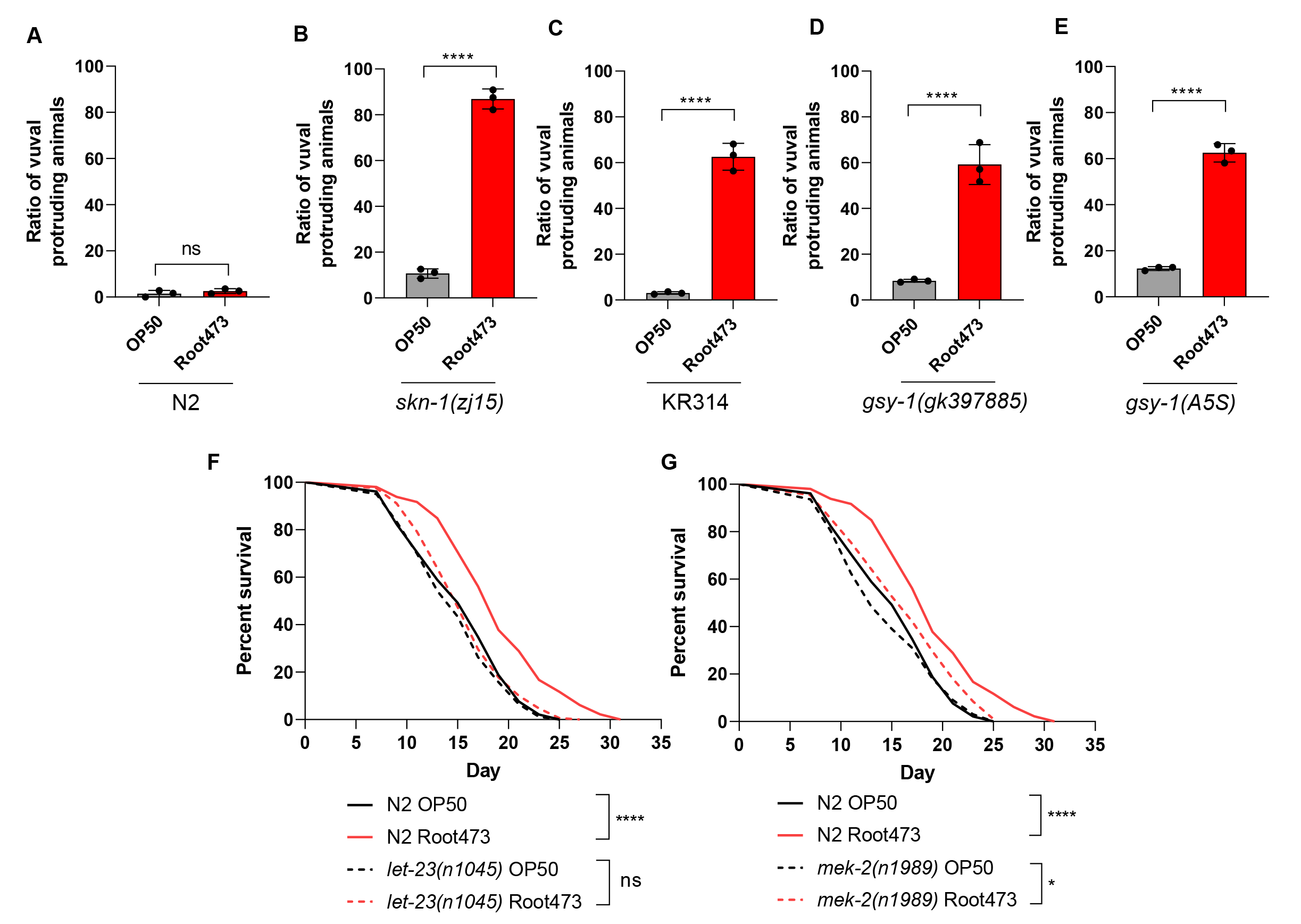

Supplement: Supplementary file 1 — Table S1: List of Arabidopsis root‐derived bacterial collections. Table S2: Medium survival changes of eight represent bacteria isolates on different genetic hosts. Table S3: Lifespan analysis of Caenorhabditis elegans wild strains on Root473 versus OP50. Table S4: Lifespan alteration on Root473 versus OP50 and lifespan alteration on OP50 of RILs. Table S5: QTL mapping data including POS and LOD score. Table S6: List of genes from QTL mapping region (II: 12148993–13479927). Table S7: Information about SNPs/indels within gsy‐1 in KR314. Table S8: RNA‐seq analysis of different regulated genes of N2 and skn‐1(zj15) on Root473 versus OP50. Table S9: Caenorhabditis elegans strains used in this study. Table S10: Source data files. Table S11: List of primers used for RT‐qPCR. Figure S1: QTL mapping of the lifespan alteration of RILs (on OP50) traits. Figure S2: Lifespan validation of candidate genes in the QTL region (II: 12148993–13479927). Figure S3: Host oxidative stress capacity determines the lifespan effects of Variovorax sp. Root473 on Caenorhabditis elegans . Figure S4: Detoxification pathways and innate immunity do not drive the genotype‐specific lifespan responses to Root473. Figure S5: Vulval integrity and impact of RAS/RAF signaling on host lifespan. Figure S6: NAC supplementation restores the mitochondrial defects and intestine integrity in skn‐1(zj15) mutants upon Root473 exposure. [file ACEL-25-e70418-s001.zip › 1_Supplementary Figure-5.tif]

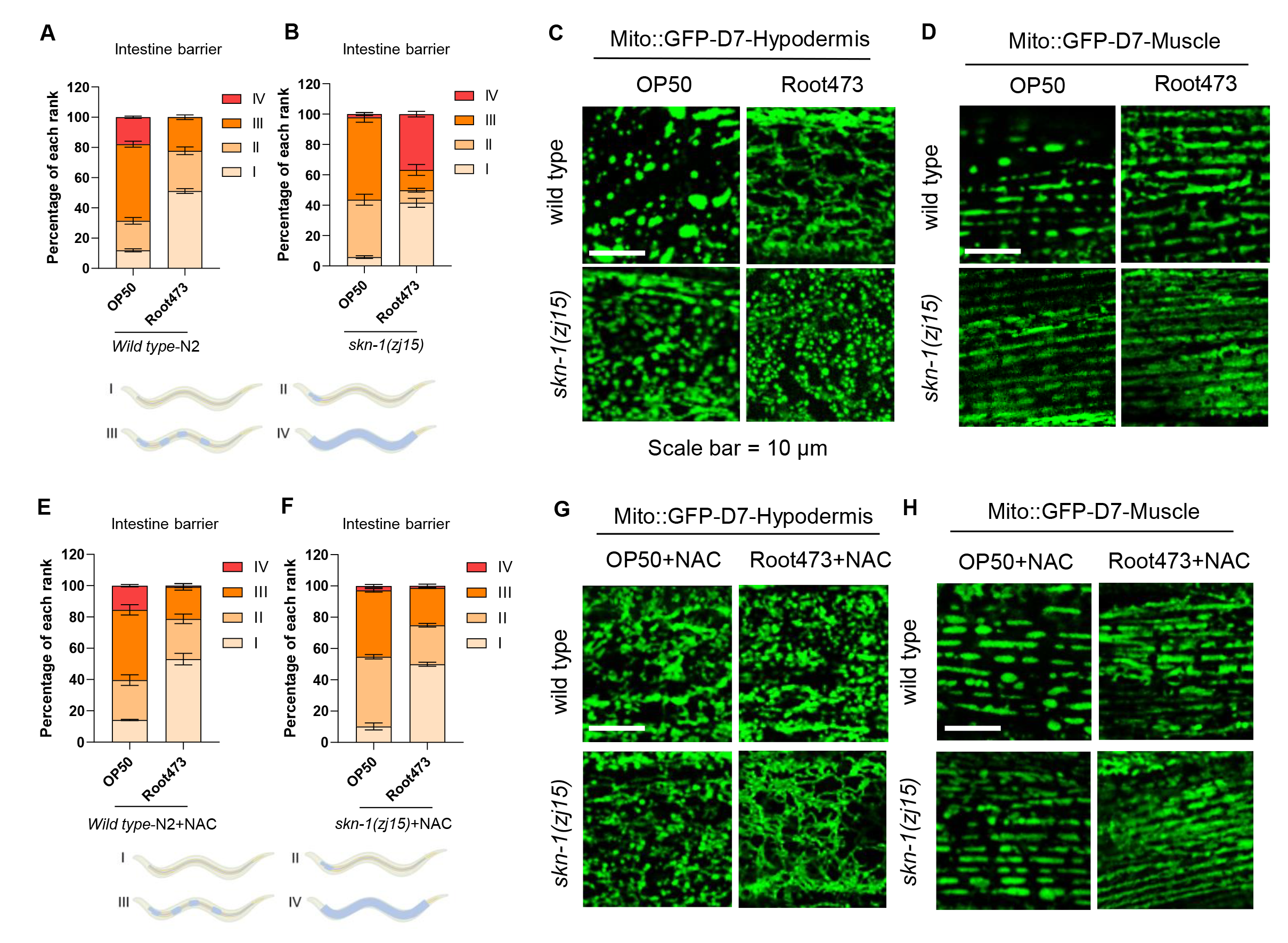

Supplement: Supplementary file 1 — Table S1: List of Arabidopsis root‐derived bacterial collections. Table S2: Medium survival changes of eight represent bacteria isolates on different genetic hosts. Table S3: Lifespan analysis of Caenorhabditis elegans wild strains on Root473 versus OP50. Table S4: Lifespan alteration on Root473 versus OP50 and lifespan alteration on OP50 of RILs. Table S5: QTL mapping data including POS and LOD score. Table S6: List of genes from QTL mapping region (II: 12148993–13479927). Table S7: Information about SNPs/indels within gsy‐1 in KR314. Table S8: RNA‐seq analysis of different regulated genes of N2 and skn‐1(zj15) on Root473 versus OP50. Table S9: Caenorhabditis elegans strains used in this study. Table S10: Source data files. Table S11: List of primers used for RT‐qPCR. Figure S1: QTL mapping of the lifespan alteration of RILs (on OP50) traits. Figure S2: Lifespan validation of candidate genes in the QTL region (II: 12148993–13479927). Figure S3: Host oxidative stress capacity determines the lifespan effects of Variovorax sp. Root473 on Caenorhabditis elegans . Figure S4: Detoxification pathways and innate immunity do not drive the genotype‐specific lifespan responses to Root473. Figure S5: Vulval integrity and impact of RAS/RAF signaling on host lifespan. Figure S6: NAC supplementation restores the mitochondrial defects and intestine integrity in skn‐1(zj15) mutants upon Root473 exposure. [file ACEL-25-e70418-s001.zip › 1_Supplementary Figure-6.tif]

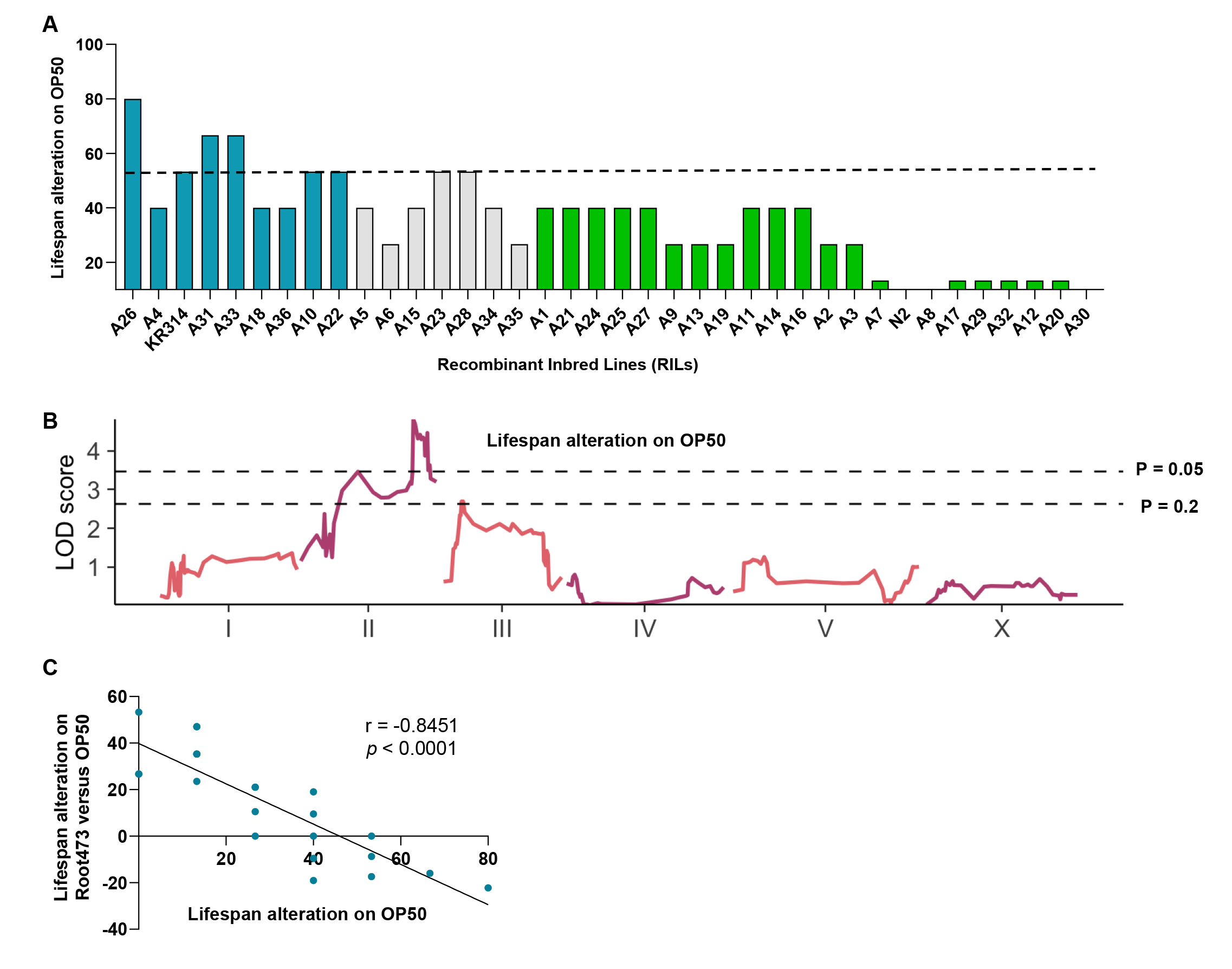

Supplement: Supplementary file 1 — Table S1: List of Arabidopsis root‐derived bacterial collections. Table S2: Medium survival changes of eight represent bacteria isolates on different genetic hosts. Table S3: Lifespan analysis of Caenorhabditis elegans wild strains on Root473 versus OP50. Table S4: Lifespan alteration on Root473 versus OP50 and lifespan alteration on OP50 of RILs. Table S5: QTL mapping data including POS and LOD score. Table S6: List of genes from QTL mapping region (II: 12148993–13479927). Table S7: Information about SNPs/indels within gsy‐1 in KR314. Table S8: RNA‐seq analysis of different regulated genes of N2 and skn‐1(zj15) on Root473 versus OP50. Table S9: Caenorhabditis elegans strains used in this study. Table S10: Source data files. Table S11: List of primers used for RT‐qPCR. Figure S1: QTL mapping of the lifespan alteration of RILs (on OP50) traits. Figure S2: Lifespan validation of candidate genes in the QTL region (II: 12148993–13479927). Figure S3: Host oxidative stress capacity determines the lifespan effects of Variovorax sp. Root473 on Caenorhabditis elegans . Figure S4: Detoxification pathways and innate immunity do not drive the genotype‐specific lifespan responses to Root473. Figure S5: Vulval integrity and impact of RAS/RAF signaling on host lifespan. Figure S6: NAC supplementation restores the mitochondrial defects and intestine integrity in skn‐1(zj15) mutants upon Root473 exposure. [file ACEL-25-e70418-s001.zip › 1_Supplementary Figure-1.tif]

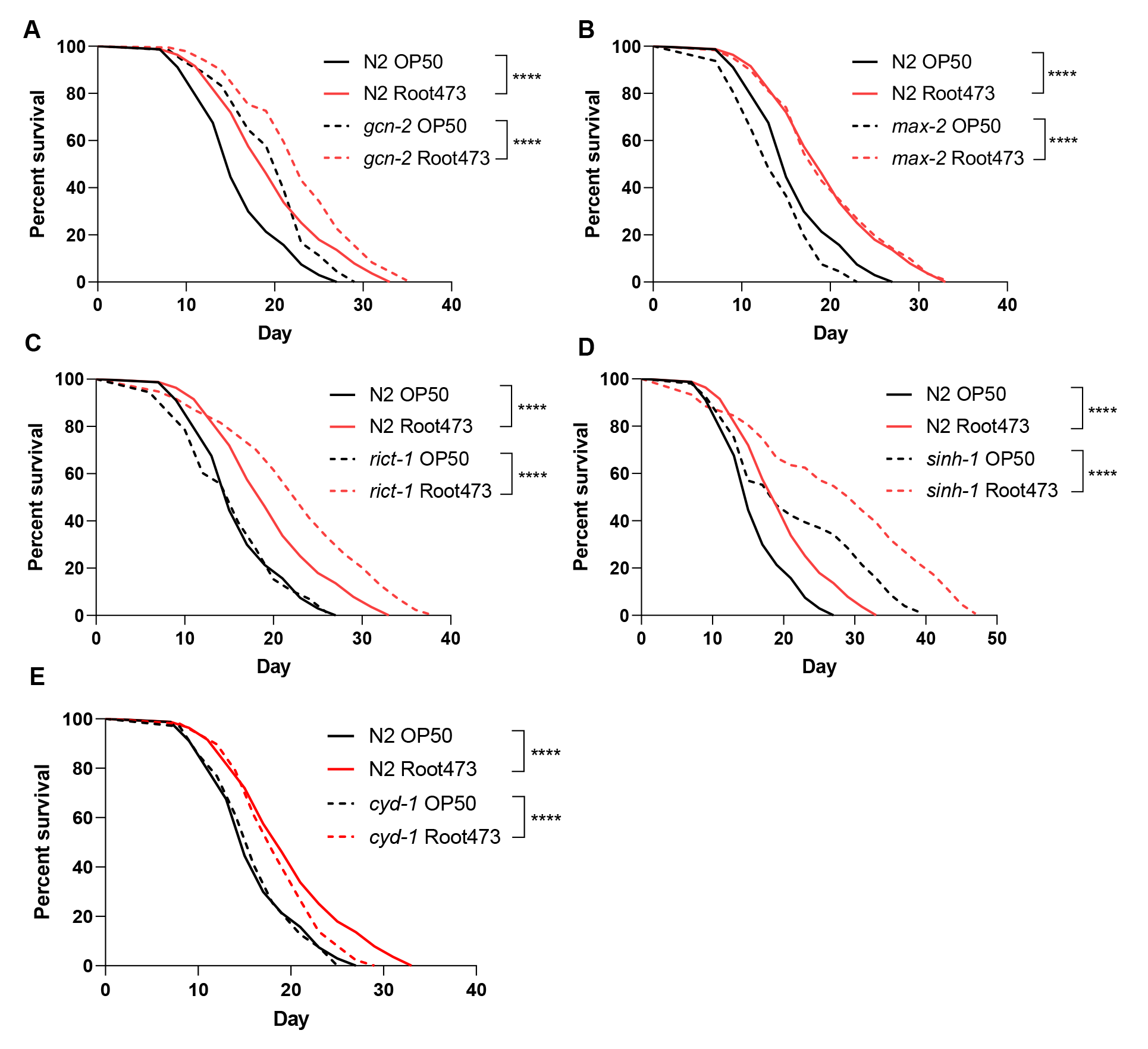

Supplement: Supplementary file 1 — Table S1: List of Arabidopsis root‐derived bacterial collections. Table S2: Medium survival changes of eight represent bacteria isolates on different genetic hosts. Table S3: Lifespan analysis of Caenorhabditis elegans wild strains on Root473 versus OP50. Table S4: Lifespan alteration on Root473 versus OP50 and lifespan alteration on OP50 of RILs. Table S5: QTL mapping data including POS and LOD score. Table S6: List of genes from QTL mapping region (II: 12148993–13479927). Table S7: Information about SNPs/indels within gsy‐1 in KR314. Table S8: RNA‐seq analysis of different regulated genes of N2 and skn‐1(zj15) on Root473 versus OP50. Table S9: Caenorhabditis elegans strains used in this study. Table S10: Source data files. Table S11: List of primers used for RT‐qPCR. Figure S1: QTL mapping of the lifespan alteration of RILs (on OP50) traits. Figure S2: Lifespan validation of candidate genes in the QTL region (II: 12148993–13479927). Figure S3: Host oxidative stress capacity determines the lifespan effects of Variovorax sp. Root473 on Caenorhabditis elegans . Figure S4: Detoxification pathways and innate immunity do not drive the genotype‐specific lifespan responses to Root473. Figure S5: Vulval integrity and impact of RAS/RAF signaling on host lifespan. Figure S6: NAC supplementation restores the mitochondrial defects and intestine integrity in skn‐1(zj15) mutants upon Root473 exposure. [file ACEL-25-e70418-s001.zip › 1_Supplementary Figure-2.tif]

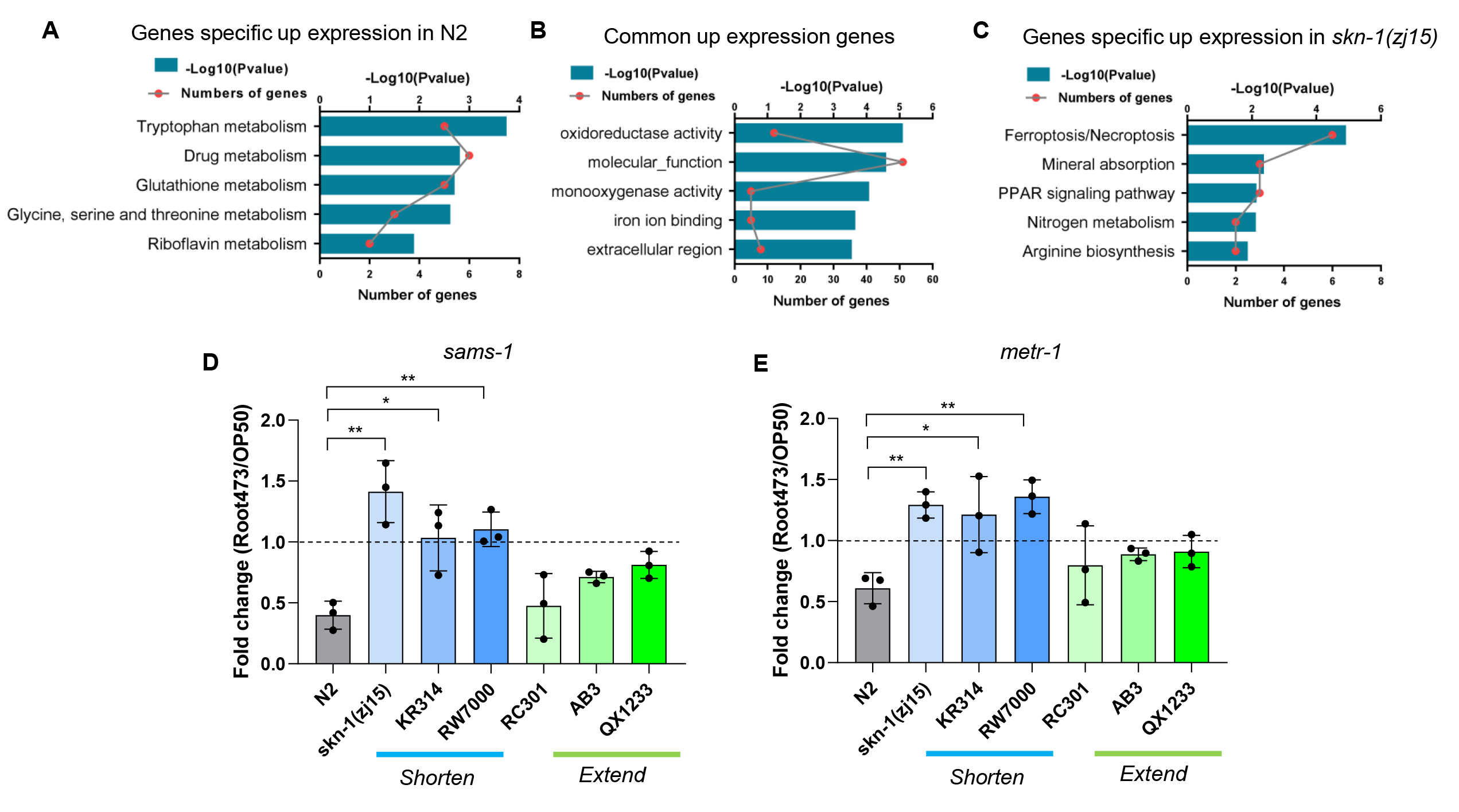

Supplement: Supplementary file 1 — Table S1: List of Arabidopsis root‐derived bacterial collections. Table S2: Medium survival changes of eight represent bacteria isolates on different genetic hosts. Table S3: Lifespan analysis of Caenorhabditis elegans wild strains on Root473 versus OP50. Table S4: Lifespan alteration on Root473 versus OP50 and lifespan alteration on OP50 of RILs. Table S5: QTL mapping data including POS and LOD score. Table S6: List of genes from QTL mapping region (II: 12148993–13479927). Table S7: Information about SNPs/indels within gsy‐1 in KR314. Table S8: RNA‐seq analysis of different regulated genes of N2 and skn‐1(zj15) on Root473 versus OP50. Table S9: Caenorhabditis elegans strains used in this study. Table S10: Source data files. Table S11: List of primers used for RT‐qPCR. Figure S1: QTL mapping of the lifespan alteration of RILs (on OP50) traits. Figure S2: Lifespan validation of candidate genes in the QTL region (II: 12148993–13479927). Figure S3: Host oxidative stress capacity determines the lifespan effects of Variovorax sp. Root473 on Caenorhabditis elegans . Figure S4: Detoxification pathways and innate immunity do not drive the genotype‐specific lifespan responses to Root473. Figure S5: Vulval integrity and impact of RAS/RAF signaling on host lifespan. Figure S6: NAC supplementation restores the mitochondrial defects and intestine integrity in skn‐1(zj15) mutants upon Root473 exposure. [file ACEL-25-e70418-s001.zip › 1_Supplementary Figure-3.tif]
