# Supplementary material for: Host Oxidative Response Capacity Determines Longevity Outcomes of Microbial Interventions
Source: Aging Cell. 2026 Feb 12;25(2):e70418. doi: 10.1111/acel.70418 (PMC12901668; doi:10.1111/acel.70418)
Supplement: Supplementary file 1 — Table S1: List of Arabidopsis root‐derived bacterial collections. Table S2: Medium survival changes of eight represent bacteria isolates on different genetic hosts. Table S3: Lifespan analysis of Caenorhabditis elegans wild strains on Root473 versus OP50. Table S4: Lifespan alteration on Root473 versus OP50 and lifespan alteration on OP50 of RILs. Table S5: QTL mapping data including POS and LOD score. Table S6: List of genes from QTL mapping region (II: 12148993–13479927). Table S7: Information about SNPs/indels within gsy‐1 in KR314. Table S8: RNA‐seq analysis of different regulated genes of N2 and skn‐1(zj15) on Root473 versus OP50. Table S9: Caenorhabditis elegans strains used in this study. Table S10: Source data files. Table S11: List of primers used for RT‐qPCR. Figure S1: QTL mapping of the lifespan alteration of RILs (on OP50) traits. Figure S2: Lifespan validation of candidate genes in the QTL region (II: 12148993–13479927). Figure S3: Host oxidative stress capacity determines the lifespan effects of Variovorax sp. Root473 on Caenorhabditis elegans . Figure S4: Detoxification pathways and innate immunity do not drive the genotype‐specific lifespan responses to Root473. Figure S5: Vulval integrity and impact of RAS/RAF signaling on host lifespan. Figure S6: NAC supplementation restores the mitochondrial defects and intestine integrity in skn‐1(zj15) mutants upon Root473 exposure. [file ACEL-25-e70418-s001.zip › 3_Supplementary File.zip!/Supplementary File/Biorender licenses/Publication License Feb-10-2026 for Figure 7.pdf]

49 Spadina Ave. Suite 200
Toronto ON M5V 2J1 Canada
www.biorender.com

Confirmation of Publication and Licensing Rights - Open Access

February 10th, 2026

Subscription Type:	*Student Plan - Academic*
Agreement number:	*SQ29CFZQKI*
Publisher Name:	*Aging Cell*
Figure Title:	*Figure 7*
Citation to Use:	*Created in BioRender. Hao, X. (2026) https://BioRender.com/d97onl0*

To whom this may concern,

This document ("Confirmation") hereby confirms that Science Suite Inc. dba BioRender ("BioRender") has granted the following BioRender user: Xusheng Hao ("User") a BioRender Academic Publication License in accordance with BioRender's Terms of Service and Academic License Terms ("License Terms") to permit such User to do the following on the condition that all requirements in this Confirmation are met:

1) publish their Completed Graphics created in the BioRender Services containing both User Content and BioRender Content (as both are defined in the License Terms) in publications (journals, textbooks, websites, etc.); and
2) sublicense such Completed Graphics under "open access" publication sublicensing models such as CC-BY 4.0 and more restrictive models, so long as the conditions set forth herein are fully met.

Requirements of User:

1) All Completed Graphics to be published in any publication (journals, textbooks, websites, etc.) must be accompanied by the following citation either as a caption, footnote or reference for each figure that includes a Completed Graphic:
"Created in BioRender. Hao, X. (2026) https://BioRender.com/d97onl0 ".
2) All terms of the License Terms including all Prohibited Uses are fully complied with. E.g. For Academic License Users, no commercial uses (beyond publication in journals, textbooks or websites) are permitted without obtaining or switching to a BioRender Industry Plan.
3) A Reader (defined below) may request that the User allow their figure to be a public template for Readers to view, copy, and modify the figure. It is up to the User to determine what level of access to grant.

Open-Access Journal Readers:

Open-Access journal readers ("Reader") who wish to view and/or re-use a particular Completed Graphic in an Open-Access journal subject to CC-BY sublicensing may do so by clicking on the URL link in the applicable citation for the subject Completed Graphic.

The re-use/modification options below are available after the Reader requests the User to adapt their

figure as a BioRender template and the User has granted such access.

1) View-Only/Free Plan Use: A Reader who wishes to only view the Completed Graphic may do so in the BioRender Services as either a BioRender Free Plan user or simply as a viewer. By becoming a BioRender Free Plan user, the Reader may view, modify and re-use the Completed Graphic as permitted under BioRender's Basic License Terms (e.g. personal use only, no publishing or commercial use permitted).
2) Re-Use/Publish with No Modifications: For any re-use and re-publication of a Completed Graphic with no modification(s) to the Completed Graphic made by the Reader, a Reader may do so by citing the original author using the citation noted above with the Completed Graphic. The Reader must also comply with the underlying License Terms which apply to the Completed Graphic as noted above (e.g. no commercial use for Academic License).
3) Re-Use/Publish with Modifications: For any re-use and re-publication of a Completed Graphic with a modification(s) made by the Reader, the Reader may do so by becoming a BioRender user themselves under either an Academic or Industry Plan, citing the original author using the citation noted above with the Completed Graphic and complying with the applicable License Terms.

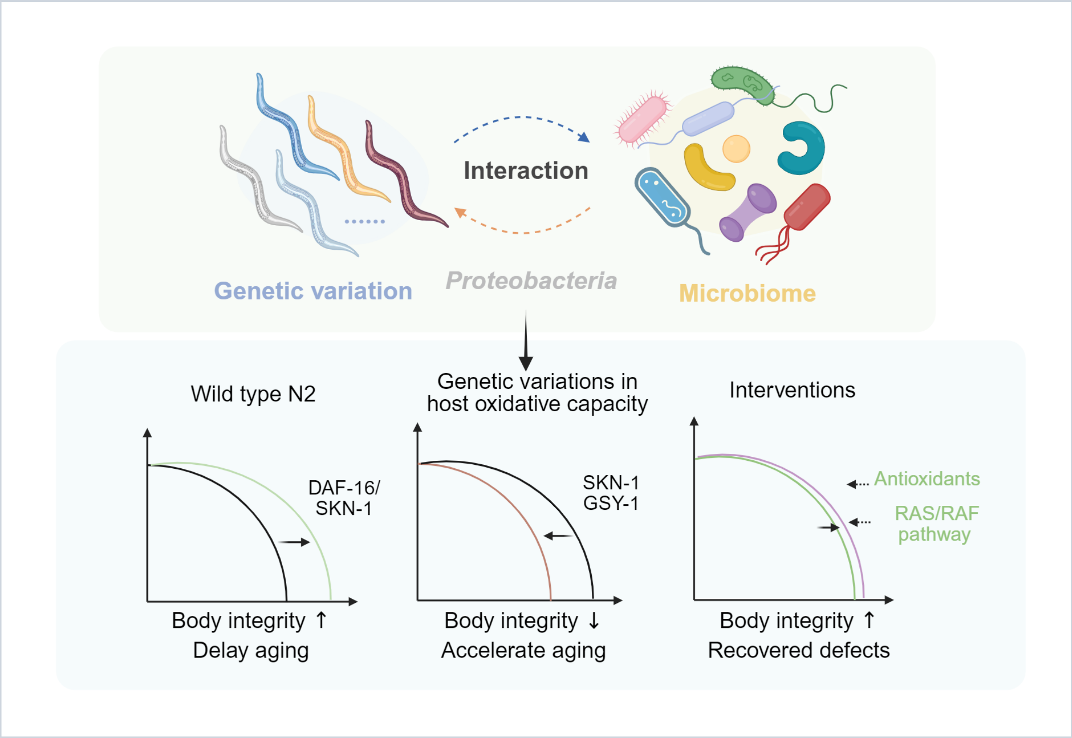

For any questions regarding this document, or other questions about publishing with BioRender, please refer to our BioRender Publication Guide, or contact BioRender Support at support@biorender.com.
